# Exploring the genetic diversity within traditional Philippine pigmented Rice

**DOI:** 10.1186/s12284-019-0281-2

**Published:** 2019-04-30

**Authors:** Edwige Gaby Nkouaya Mbanjo, Huw Jones, Xavier Greg Isaguirre Caguiat, Socorro Carandang, John Carlos Ignacio, Marilyn Cruz Ferrer, Lesley Ann Boyd, Tobias Kretzschmar

**Affiliations:** 1 International Rice Research Institute (IRRI), Pili Drive, 4031 Los Baños, Laguna Philippines; 2 National Institute of Agricultural Botany (NIAB), Huntingdon Road, Cambridge, CB3 0LE UK; 3 Philippine Rice Research Institute (PhilRice), Maligaya, Science City of Muñoz, 3119 Nueva Ecija, Philippines; 4 Southern Cross University, 1 Military Road, East Lismore, 2480 NSW Australia

**Keywords:** Genetic diversity, Population structure, Pigmented rice, Single nucleotide polymorphisms

## Abstract

**Background:**

The wild ancestors of domesticated rice had red seed, white rice being the result of a mutation in the rice domestication gene *Rc*. Many pigmented rice landraces are still grown by ethnic communities for their nutritional and cultural value. This study assesses the genetic diversity in a collection of pigmented rice accessions from the Philippines.

**Results:**

We undertook an analysis of the genetic and colour variation in a collection of 696 pigmented rice accessions held at PhilRice in the Philippines. The collection was reduced to 589 genotypes after removal of accessions with limited passport data or with low SNP marker call rates. Removal of duplicate genotypes resulted in a final, core collection of 307 accessions, representing all administrative districts of the Philippines, and composed predominately of *japonica* and *indica* sub-species. No genetic structure was observed in the core collection based on geographic origin. A pairwise comparison of accessions by region indicating that both local and long-distance exchange of rice accessions had occurred. The majority of the genetic variation was within regions (82.38%), rather than between regions (10.23%), with the remaining variation being within rice accession variance (7.39%). The most genetically diverse rice accessions originated from the Cordillera Administrative Region (CAR) in the far north of the Philippines, and in the regions of Davao and Caraga in the southeast. A comparison with pigmented rice accessions from the neighbouring countries Taiwan, Laos, China and India revealed a close relationship between accessions from Taiwan, supporting the hypothesis of southward diffusion of Austronesians from Taiwan to the Philippine. The 14-bp deletion within the gene *Rc*, known to result in loss of red pigmentation, was found in 30 accessions that still had coloured pericarps. Multi-spectral phenotyping was used to measure seed geometric and colour-appearance traits in 197 accessions from the core collection. The purple and variable purple rice accessions had the lowest values for the seed colour parameters - lightness (L*), intensity, saturation, a* (green – red; redness) and b* (blue – yellow; yellowness).

**Conclusion:**

These pigmented rice accessions represent a diverse genetic resource of value for further study and nutritional improvement of commercial rice varieties.

**Electronic supplementary material:**

The online version of this article (10.1186/s12284-019-0281-2) contains supplementary material, which is available to authorized users.

## Background

Rice (*Oryza sativa*) is a staple food for over half of the world’s population. While rice can provide up to 50% of the daily calorie intake, white rice is a poor source of vitamins and minerals, and is associated with malnutrition among communities where rice is the predominant food (Dipti et al. 2012; Muthayya et al. 2014; World Rice Production 2019). Un-milled rice is a richer source of nutritionals, and several attempts have been undertaken to make un-milled rice softer and more palatable, thereby improving consumer acceptance (Raghuvanshi et al. 2017; Mardiah et al. 2018). An alternative solution would be to identify rice genotypes able to accumulate higher levels of nutritionals within the endosperm, improving the nutritional value of white rice.

The Philippines is the world’s eighth largest rice producer, with a share of 2.8% of total global production (Sharma 2017; World Rice Production 2019). However, it is also the second largest rice net importer in the world, with 20% of domestic rice consumption being sourced from imports (Wailes and Chavez 2012). As rice is a major component of the Philippine diet, increasing the nutritional value of rice varieties would greatly benefit the health of the population. However, high market demand for white rice has resulted in depletion of pigmented varieties (Ahuja et al. 2007). Most pigmented rice varieties are low yielding, being grown for local markets (Wickert et al. 2014; Mau et al. 2017).

Rice can produce grain with brown, red, purple and even black pericarps. Black and purple pericarps are the result of accumulation of anthocyanin, while red pericarps are due to proanthocyanidins (Gunaratne et al. 2013; Samyor et al. 2017). Pigmented rice varieties tend to have a higher protein content with a well-balanced amino acid composition, a better glycemic index and higher levels of fats, fiber and vitamin E (tocopherols and tocotrienols) (Gunaratne et al. 2013; Hedge et al. 2013; Kushwaha 2016). They also exhibit strong antioxidant and free radical scavenging capacity due to high levels of phenolic compounds (e.g. anthocyanin, proanthocyanidin and phenolic acids; Samyor et al. 2017).

The wild ancestor of rice had red grain. White grained rice resulted from a loss of function mutation (14-bp deletion – *rc* allele) within exon 6 of the *Rc* gene; a basic Helix-Loop-Helix (bHLH) protein (Furukawa et al. 2006; Sweeney et al. 2006). The intensity of the red colouration is determined by a complementary interaction between *Rc* and a second gene, *Rd;* a dihydroflavonol-4-reductase (Furukawa et al. 2006; Sweeney et al. 2006). While *Rc* is responsible for the accumulation of proanthocyanidines in the pericarp, *Rd* regulates the level of accumulation. The regulation of anthocyanins in rice pericarps is less well understood. A *C-S-A* gene model has been proposed to control pigmentation in rice hulls (Sun et al. 2018), where *C1* encodes a R2R3MYB transcription factor that alone results in brown rice hulls, but in combination with *A1*; a dihydroflavonol reductase, produces purple hulls. However, *C1* is not involved in pigmentation in rice pericarps. *S1* encodes a bHLH protein and is required for both purple and brown hull pigmentation. Other genes reported to be involved in rice grain pigmentation include *Kala1, Kala3* and *Kala4* (Maeda et al. 2014), *Pb* (Caixia and Qingyao 2007; Rahman et al. 2013) and *Pp* (Rahman et al. 2013). Ectopic expression of the *Kala4 bHLH* gene, caused by a rearrangement in the promotor region, was shown to result in black grained rice (Oikawa et al. 2015). Kinoshita (1995) reported the involvement of 26 genes in anthocyanin production.

*O. sativa* is composed of two sub-species, *japonica* and *indica*. The *indica* sub-species has been divided into two sub-populations, *indica* and *aus*, while *japonica* divides into tropical *japonica*, temperate *japonica* and aromatic sub-populations (Garris et al. 2005). In addition to the widely accepted five major rice sub-populations a new group, *rayada* was reported by Wang et al. (2014). Population structure and genetic diversity analyses of the 3 k SNP-Seek data revealed a higher level of diversity within *indica* than *japonica,* and identified additional sub-populations in both sub-species (Wang et al. 2018). Accessions that fell between groups, classified as admixed, have also been reported (Wang et al. 2014, 2018).

Few studies of genetic diversity within pigmented rice varieties have been reported, and involve small numbers of genotypes and markers (Patel et al. 2014; Ahmad et al. 2015; Ashraf et al. 2016). The most extensive of these involved 42 pigmented rice accessions collected from across the far east, many from the Philippines. SSR markers grouped these genotypes according to geographic origin (Ahmad et al. 2015). The studies involving Indian pigmented rice accessions (19 and 16 accessions, respectively) had similarity indexes ranging from 0.56 to 0.95 (Patel et al. 2014) and 0.00 to 0.647 (Ashraf et al. 2016).

Local landrace collections, such as the Philippine traditional pigmented rice collection investigated in this study, represent a valuable genetic resource for rice breeding. Comprehensive evaluation of the genetic diversity within such collections is essential to utilize the nutritional value of these pigmented rice genotypes in the development of high yielding, pigmented rice varieties (Roy et al. 2015). Proper genetic characterization can also aid with geographical indication of rice accessions, required for the protection of ownership by Philippine indigenous communities, and ensure authenticity and purity of varieties in the market.

In this study we assess the genetic diversity and population structure within a collection of Philippine traditional pigmented rice accessions held at the PhilRice Genebank using a rice Illumina Infinium® II SNP genotyping array. The genetic relationship between the Philippine rice accessions is compared by region of origin, and to pigmented rice accessions from the neighbouring countries of Taiwan, China and India. We further use the SNP data to select markers that define individual accessions within a selected, core collection of the Philippine traditional pigmented rice. An extensive multi-spectral analysis of the pigmented seed, looking at seed shape and colour, is undertaken, along with a screen for the *Rc* 14-bp del mutation known to confer loss of red pigmentation. Taken all together this study provides a comprehensive examination of the genetic potential within a collection of traditional, pigmented Philippine rice accessions.

## Results

### Primary assessment of the Philippine pigmented Rice accessions and assignment to Rice varietal groups

**Fig. 1:**
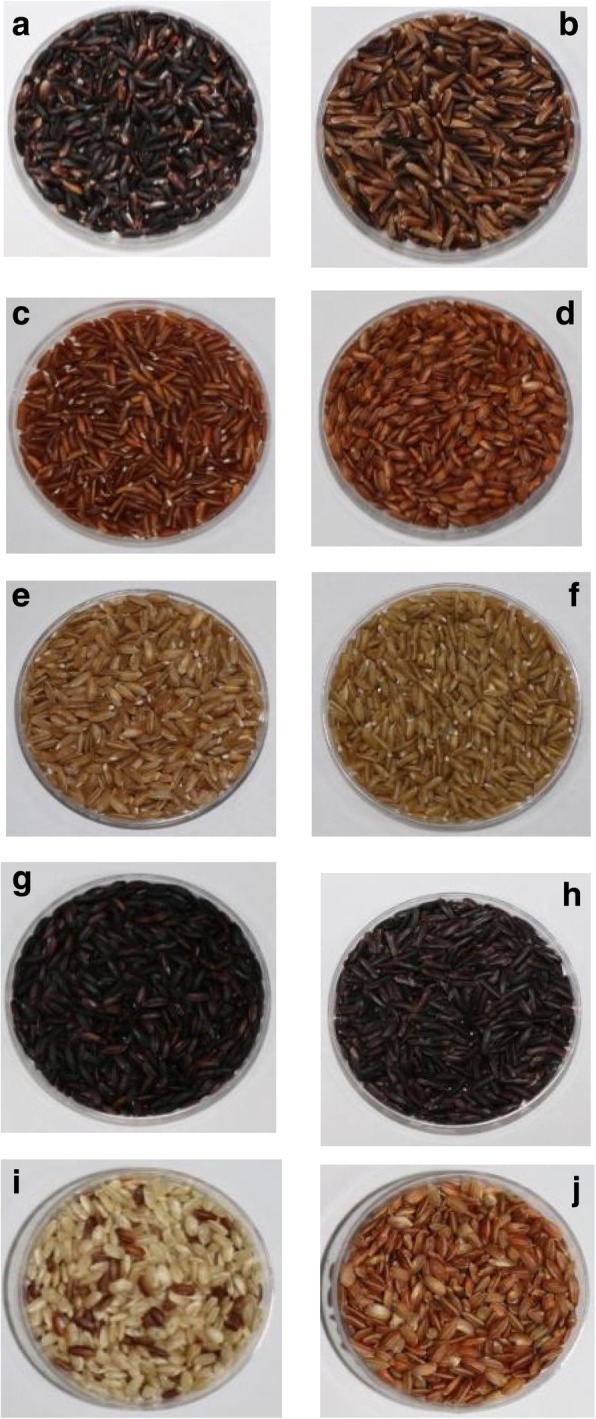
Diversity in seed pericarp colour among the Philippine pigmented rice accessions. Samples of rice pigmentations; a and b = variable purple, c and d = red, e and f = brown, g and h = dark purple or black, i and j = mixed (red/white)

Visual assessment of the seed colour of the original 696 Philippine rice accession, using bioversity indicators for pericarp colour (Bioversity International et al. 2007) qualified 81.47% as red, 7.61% as variable purple, 4.89% as purple, 1.43% as white, 0.29% as brown, 1.72% with mixed seed colour and 2.59% with undefined colour (Fig. 1).

**Fig. 2:**
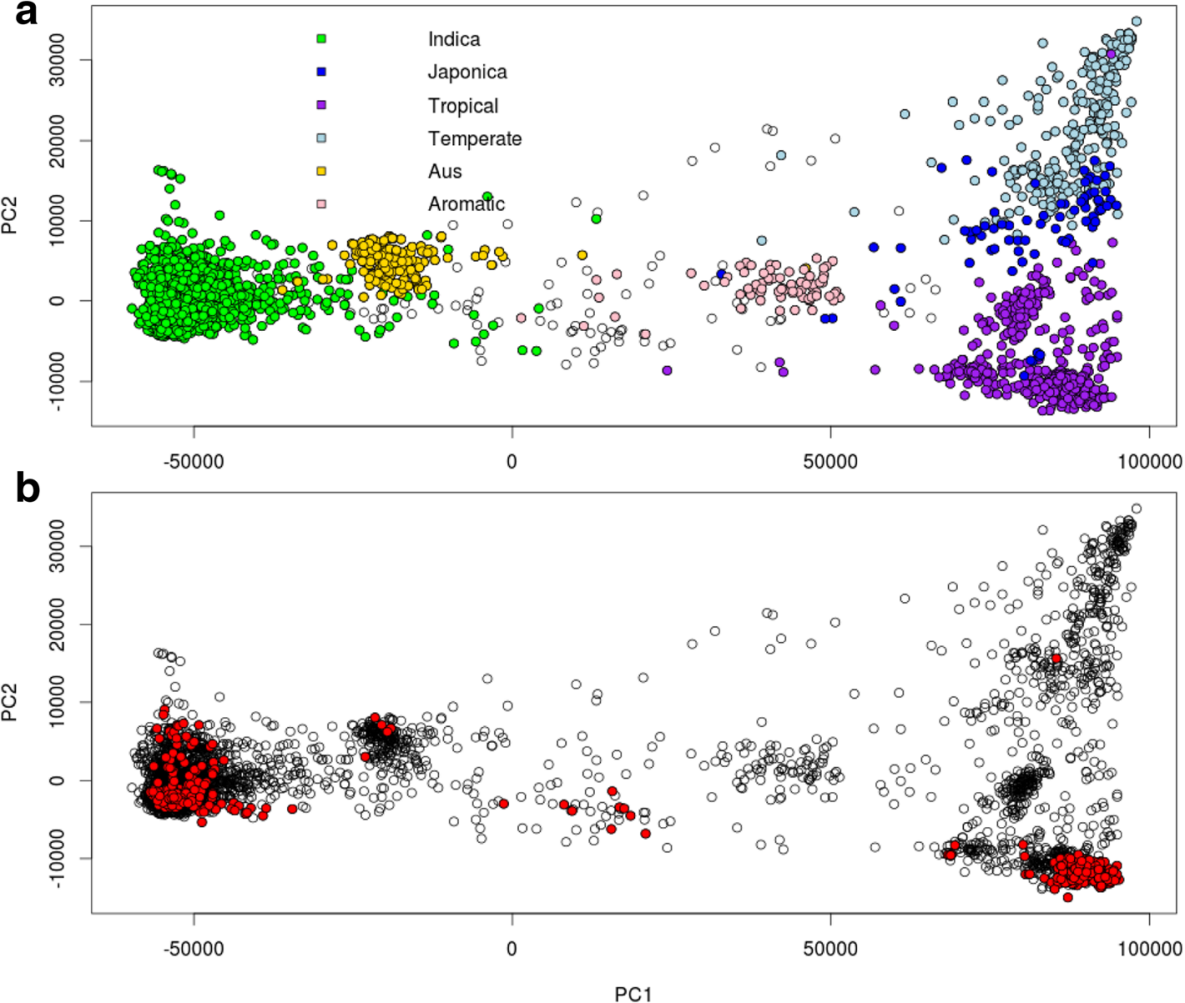
**a** Principal component analysis (PCA) of the rice 3 k SNP Seek data set (Mansueto et al. 2017). The main rice sub-groups are indicated by different colours; blue-*japonica*, green-*indica*, purple-tropical, pink-aromatic, light blue-temperate and yellow-*aus*. **b** PCA of the 696 Philippine pigmented rice accessions (red dots) overlayed on the PCA of the rice 3 k SNP Seek set. The additive relationship matrix was calculated for each rice data set in rrBLUP, using the R package rrBLUP (Endelman, 2011), and used as input to the PCA

Overlaying the 696 Philippine pigmented rice accessions on a PCA analysis of the 3027 rice genomes from the SNP-Seek database (Mansueto et al. 2017) (Fig. 2) indicated that the majority of the Philippine accessions belonged to the *japonica* and *indica* subgroups. While a combined cluster analysis indicated that 60.78% of the Philippine rice accessions were *japonica* and 36.93% *indica* types (Additional file 1: Figure S1). No Philippine pigmented rice accessions clustered with the *aromatic* rice subgroup.

Philippine pigmented rice accessions of unknown origin (13.79%) or with a SNP marker call rate of < 0.90 were removed from the collection, leaving 589 accessions for subsequent analysis, along with 1536 SNP markers that had passed quality control.

### Genetic relationship between the Philippine pigmented Rice accessions

Cryptic relatedness analyses of the 589 rice accessions, using the 1536 SNP markers that had passed the quality control checks, revealed the presence of duplicate rice genotypes (accession sample genotypes with an estimated Probability of Identity (estimated PI) = 1; Additional file 1: Figure S2). These duplicated rice accessions were removed, leaving a single accession representative of each genotype, forming a core collection of 307 accessions. This core collection was subsequently used in all further analyses (Additional file 1: Figure S3; Additional file 2: Table S1). A weak or absent relatedness was detected between some rice accession pairwise comparisons (estimated PI < 0.5), while other rice accessions were related at sibling (estimated PI ≥0.5), or half-sib level (0.25 ≤ estimated PI < 0.5). Geographically close regions with a small number of accessions were merged, and a new region defined strictly for the ease of further analyses. Rice accession sample size in the new defined regions ranged from 11 in the Cagayan Valley and Bicol Region subgroup, to 45 rice accessions in the Cordillera Administrative Region (CAR) subgroup (Additional file 3: Table S9).

### Population structure within the Philippine pigmented Rice accessions

**Fig. 3:**
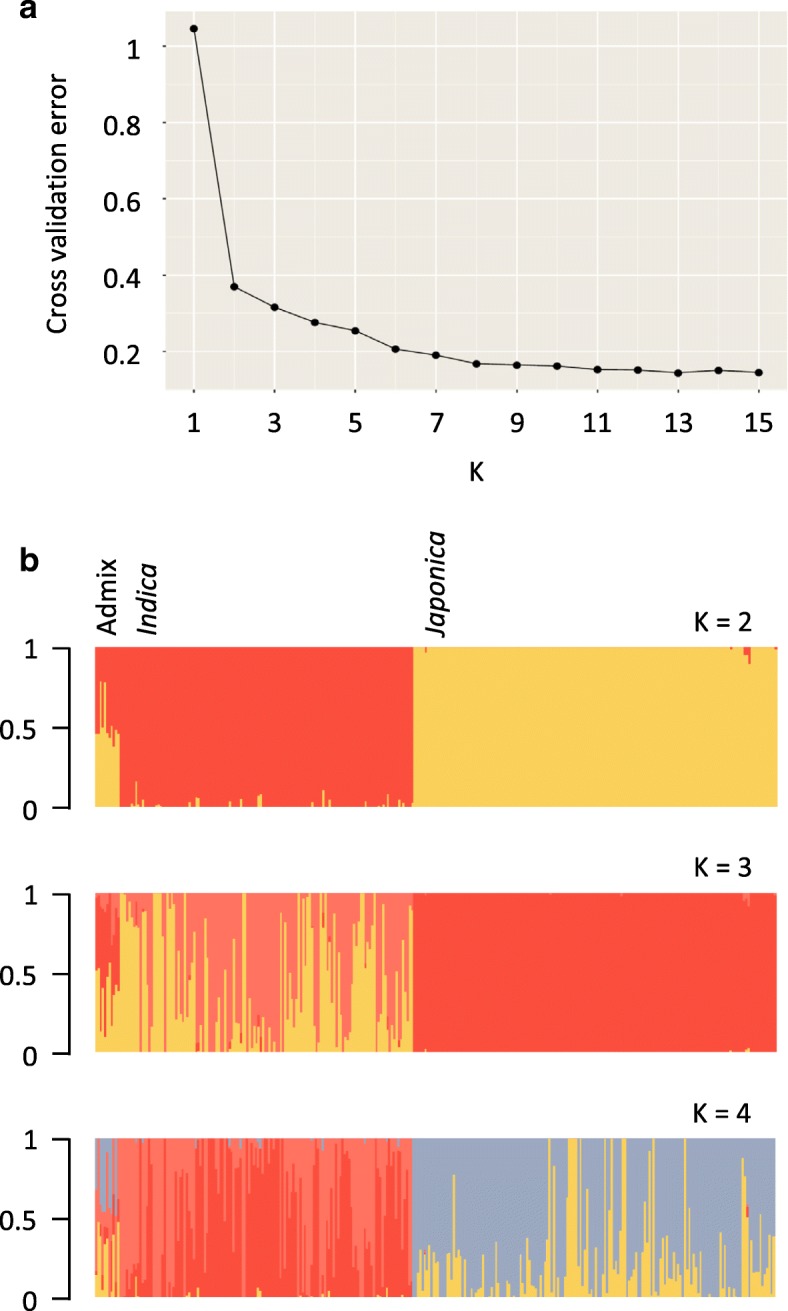
Population structure within the 307 core Philippine pigmented rice accessions, showing partitioning into two main populations. **a** Cross-validation plot. Cross-validation error is shown on the Y-axis (vertical) and the number of hypothetical populations on the X-axis (horizontal). **b** Individual ancestry inferred with ADMIXTURE. Each individual is represented by a vertical line partitioned by colour. The proportion of the colour making up each vertical line represents the proportion contributed by the ancestral population. The best supported clustering (K = 2) divided the 307 rice accessions into two main groups, corresponding to *indica* and *japonica* rice types. With increased K values (K = 3 and 4) additional substructure within each cluster was observed

**Fig. 4:**
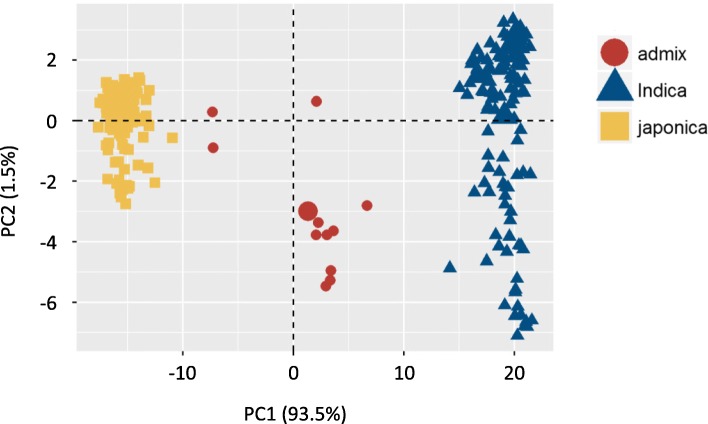
Principal component analysis of 307 core Philippine pigmented rice accessions. The additive relationship matrix was calculated for the 307 core Philippine pigmented rice accessions in rrBLUP using the R package rrBLUP (Endelman, 2011) and used as input to the PCA. The 307 accessions were separated into two main groups, *indica* (blue) and *japonic*a (yellow)

The 307 unique rice accessions were clearly separated into two main clusters corresponding to *indica* and *japonica* rice types, results being consistent between the model-based clustering analysis implemented in ADMIXTURE (Fig. 3), the phylogenetic analysis (Additional file 1: Figure S4) and the principal component analysis (Fig. 4). Only 3.58% of the accessions were classified as admix, while 43.0% and 53.42% were classified as *indica* and *japonica*-type, respectively. Admix accessions were scattered in the center of the PCA plot (Fig. 4), the first two principal components accounting for 93.5% and 1.5%, respectively of the total variance in the dataset. No structuralisation was observed based on geographic origin (Additional file 1: Figure S5). No clear clustering of rice accessions, based on geographical origin, being observed in the PCA. However, slight discrepancies were seen between the model-based cluster and the unrooted neighbour-joining (NJ) analyses. One accession (PTRV121), that had less than 80% probability of a single group membership using the maximum likelihood estimates, was classified as admix-type, while phylogenetic analyses, using the NJ method, assigned the accession to the *japonica* sub-species. This slight discrepancy between the clustering methods may be due to differences in thresholds used to assign each accession to a rice varietal group. Structure analyses performed independently on the *indica* and *japonica* sub-species data sets supported the substructure within each group (Additional file 1: Figures S6 and S7). When the SNP-based PCA was annotated based on pericarp colouration, the pigmentation of the pericarp was observed in both *japonica* and *indica* accessions (Additional file 1: Figure S8).

### Estimates of genetic diversity within the Philippine pigmented Rice accessions

Assessment of genetic diversity indicators within the 307 unique Philippine pigmented rice accessions (He = 0.35) revealed a higher level of genetic diversity within the admix group (He = 0.35) compared to the *indica* (He = 0.26) and *japonica* (He = 0.21) sub-species (Wilcoxon signed rank test, *p* < 0.05). A similar pattern was observed between the groups for allele richness (Table 1). Genetic diversity at the regional level ranged from 0.26 to 0.34 (He). The most diverse group of rice accessions were from the CAR and Mindanao 3 regions (Davao and Caraga) (He = 0.34) (Table 1).

**Table 1:** Summary statistics of genetic diversity indicators comparing the Philippine pigmented rice accessions by rice subspecies and between regions of origin

		A	%	Ar ± SD	Ho ± SD	He ± SD	FIS	FIS_Low	FIS_High
Subspecies	Admix	3009	97.95	1.92 ± 0.21	0.3 ± 0.17	0.35 ± 0.14	0.16	−0.20	0.49
	*Indica*	2788	90.76	1.64 ± 0.41	0.02 ± 0.072	0.26 ± 0.19	0.91	0.87	0.94
	*Japonica*	2531	82.39	1.54 ± 0.45	0.01 ± 0.07	0.21 ± 0.97	0.95	0.94	0.95
Regions	NCR	2791	90.85	1.7 ± 0.37	0.06 ± 0.09	0.26 ± 0.18	0.78	0.57	0.95
	CAR	3046	99.15	1.86 ± 0.22	0.03 ± 0.07	0.34 ± 0.15	0.90	0.82	0.97
	Ilocos Region	3000	97.66	1.75 ± 0.28	0.02 ± 0.07	0.27 ± 0.16	0.91	0.80	0.97
	Cagayan Valley	2826	91.99	1.74 ± 0.35	0.03 ± 0.08	0.28 ± 0.17	0.89	0.73	0.98
	Central Luzon	3052	99.35	1.84 ± 0.22	0.03 ± 0.07	0.32 ± 0.14	0.92	0.84	0.97
	Calabarzon	2993	97.43	1.81 ± 0.26	0.01 ± 0.07	0.31 ± 0.15	0.96	0.95	0.97
	MIMAROPA	2995	97.49	1.74 ± 0.29	0.01 ± 0.06	0.27 ± 0.16	0.96	0.94	0.97
	Bicol Region	2956	96.22	1.84 ± 0.27	0.05 ± 0.08	0.33 ± 0.15	0.86	0.72	0.96
	Visayas	2978	96.94	1.83 ± 0.27	0.04 ± 0.07	0.32 ± 0.15	0.88	0.70	0.98
	Mindanao1	3016	98.18	1.79 ± 0.25	0.01 ± 0.07	0.29 ± 0.15	0.95	0.92	0.97
	Mindanao2	2994	97.46	1.84 ± 0.26	0.05 ± 0.08	0.32 ± 0.14	0.85	0.66	0.96
	Mindanao3	3006	97.85	1.85 ± 0.24	0.01 ± 0.06	0.34 ± 0.14	0.97	0.96	0.98

Total number of alleles observed across SNP marker loci (A). Total observed alleles per locus as a percentage of population sample (%).Mean allele richness (Ar). Observed heterozygosity across loci (Ho). Expected heterozygosity across loci (He). Inbreeding coefficient (FIS) showing the 95% confidence intervals (FIS_Low and FIS_High). SD, standard deviations

When the *indica* and *japonica* accessions were analyzed separately, the *japonica* accessions exhibited a lower level of polymorphism than the *indica* accessions, with a mean He value of 0.18 across regions versus a mean He value of 0.22 for the *indica* accessions (Additional file 3: Table S10). Bicol Region and National Capital Region (NCR) (He = 0.24) were the most diverse regions in the *indica* group, while CAR and Mindoro, Marinduque, Romblon and Palawan (MIMAROPA) (He = 0.21) exhibited the least diversity. There was greater variation in genetic diversity across regions in the *japonica* group, He values ranging from 0.15 in Visayas to 0.2 in Central Luzon, MIMAROPA and Mindanao1 (Northern Mindanao and ARMM).

The inbreeding coefficient values (FIS) were 0.16, 0.91 and 0.95 for admix, *indica* and *japonica*, respectively (Table 1). The high FIS values for the *indica* and *japonica* accessions indicates a high level of within group relatedness. Within regions FIS values were generally high, positive and significant, ranging from 0.78 in NCR to 0.97 in Mindanao3. The *indica* accessions from the Bicol Region exhibited the lowest FIS value (Additional file 3: Table S10). At the level of individual rice accessions the heterozygosity rate was low in most accessions, with only a few heterogeneous accessions being detected (Additional file 1: Figure S9**)**.

### Population differentiation within the Philippine pigmented Rice accessions

**Fig. 5:**
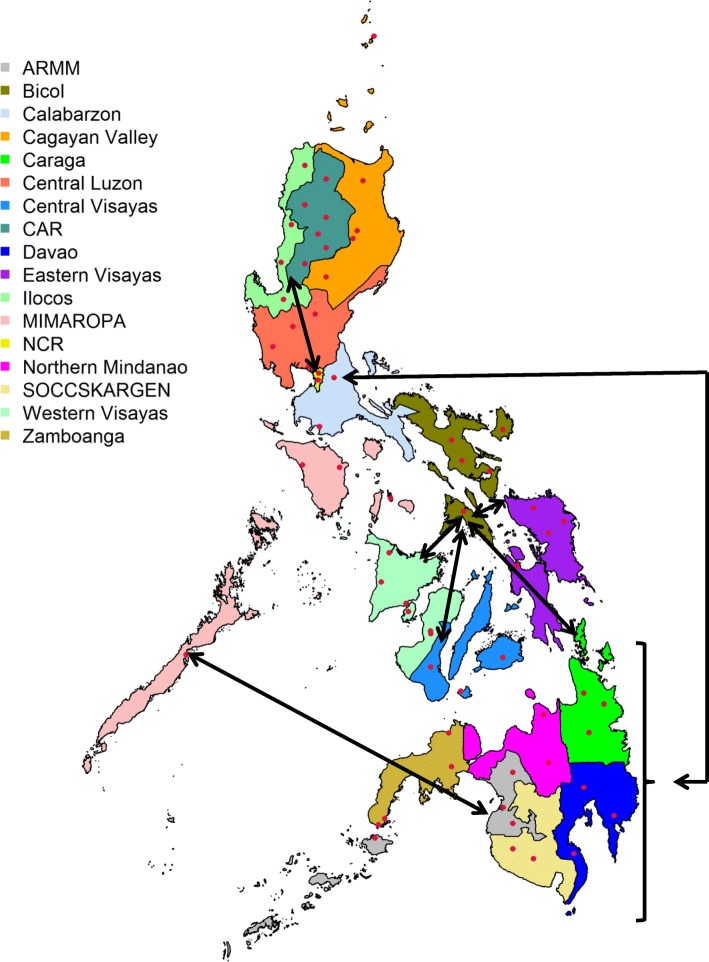
Geographical origin of Philippine pigmented rice accessions. Accessions were collected in 63 provinces, as indicated by red circles. The arrows show the predicted movement of rice accessions as determined by *F*_ST_ analyses. ARMM = Autonomous Region of Muslim Mindanao, CAR = Cordillera Administrative Region, MIMAROPA = Mindoro, Marinduque, Romblon and Palawan, NCR = National Capital Region, SOCCSKSARGEN = South Cotabato, Cotabato City, North Cotabato, Sultan Kudarat, Sarangani and General Santos City

An *F*_ST_ test was used to assess genetic differentiation between all possible groups based on allele discrepancies (Additional file 2: Table S2**)**. Genetic differentiation was high between the *indica* and *japonica* groups (*F*_ST_ = 0.50). High structuralization was also observed between the *indica* and admix groups (*F*_ST_ = 0.21), and between the *japonica* and admix groups (*F*_ST_ = 0.21). When considering the geographic origin of the rice accessions (Fig. 5) the pairwise *F*_ST_ values ranged from very low (*F*_ST_ = − 0.0099, indicating no difference between the regions, probably due to high rice genotype migration) to high (*F*_ST_ = 0.33, indicating considerable genetic differentiation between regions).

Accessions from the Ilocos Regions were highly differentiated (*F*_ST_: 0.17 to 0.24) from accessions originating from Mindanao 2, Mindanao 3, Visayas, Calabarzon and Central Luzon, and even higher for accessions collected from Mindanao1, MIMAROPA and the Cagayan Valley (*F*_ST_ > 0.28), indicating genetic separation of rice accessions from these regions. A similar observation was reported between rice accessions from NCR versus Mindanao1, Mindanao 2, Mindanao 3, Central Luzon, Calabarzon, MIMAROPA, Cagayan Valley and Visayas (*F*_ST_: 0.15 to 0.23). However, the average genetic differentiation over all regions was 0.09, showing that a high proportion of the variation was due to within region variation.

Low *F*_ST_ values were seen when comparing the neighbouring regions of Mindanao 1 (Zamboanga Peninsula/ Northern Mindanao/ ARMM (Autonomous Region of Muslim Mindanao)), Mindanao 2 (SOCCSKSARGEN (South Cotabato, Cotabato City, North Cotabato, Sultan Kudarat, Sarangani and General Santos City) and Mindanao 3 (Davao Region/Caraga), and the neighboring regions of Calabarzon and Central Luzon (*F*_ST_ = 0.0099) indicating seed exchange between these regions. However, long distance transfer of rice seed was apparent between Calabarzon and Mindanao 1 (*F*_ST_ = 0.0099), Mindanao 2 (*F*_ST_ = 0.0099) and Mindanao 3 (*F*_ST_ = 0.0077), and between MIMAROPA and Mindanaol 1 (*F*_ST_ = 0.0054). Rice seed movement was also apparent between Bicol and the regions of Central, Eastern and Western Visayas (*F*_ST_ = 0.0084). Seed from Bicol may have found its way to Mindanao 3 (*F*_ST_ = 0.0059), via Visaya (Visayas – Mindanao 3 *F*_ST_ = 0.0024). In addition there was very little genetic differentiation (*F*_ST_ = 0.0006) between rice accessions from the regions of Ilocos and NCR.

Looking just at the *indica* group of Philippine pigmented rice accessions, pairwise *F*_ST_ comparisons between regions ranged from little or no genetic variation (*F*_ST_ = − 0.04), to reasonable levels of genetic variation (*F*_ST_ = 0.19). Significant differences were seen between *indica* accessions from the Ilocos region, and accessions from the MIMAROPA (*F*_ST_ = 0.15) and Mindanao1 regions (*F*_ST_ = 0.17) (Additional file 2: Table S3). Differentiation was also observed within the *indica* group of accessions from CAR and Mindanao 1, Mindanao 2 and MIMAROPA (*F*_ST_: 0.16 to 0.19). Considering only the *japonica* rice accessions, greatest genetic differentiation was seen between accessions from Visayas and Cagayan Valley (*F*_ST_ = 0.21; Additional file 2: Table S4). The average genetic differentiation over all regions, in both rice groups, was slightly lower (mean *F*_ST_ *indica* = 0.07, mean *F*_ST_ *japonica* = 0.08) than the average genetic differentiation when all rice accessions were analyzed together, again indicating that most of the genetic variation was due to within region variation.

### Analyses of molecular variance within the Philippine pigmented Rice accessions

An analysis of molecular variance (AMOVA; Table 2) indicated that 47.13% of the total genetic variation occurred between the *indica*/*japonica*/admix groupings, while within group differences accounted for 46.96% of the variation. Variation within the rice accessions, representing residual heterozygosity, accounted for 5.91% of the total genetic variation. After removing the admix accessions, the between group molecular variance increased to 50.14%, while the within group variation fell to 46.31% (Additional file 3: Table S11). When the rice accessions were grouped based on their geographic origin, a substantial part of the genetic variation was within the regions (82.38%), very little variation being observed between regions (10.23%); substantiating the *F*_ST_ analyses. The remaining variation was accounted for by within rice accession variance (7.39%). When *indica* and *japonica* accessions were analyzed separately, the AMOVA results again showed that most of the variation occurred within regions, with the *japonica* and *indica* rice groups showing 87.18% and 84.05% variation, respectively within geographical regions (Additional file 3: Table S11).

**Table 2:** Analysis of molecular variance of the Philippine pigmented rice accessions

	Source of variation	Degree of freedom	Sum of squares	Mean sum of square	Estimate variance	% of variation
Subspecies	Between rice varietal types	2	53,146.6	26,573.3	161.7	47.1
	Between accessions within varietal types	304	104,110.4	342.5	161.1	46.9
	Within rice accessions	307	6225.4	20.3	20.3	5.9
	Total	613	163,482.5	266.7	343.1	100
Regions	Between regions	11	20,760.3	1887.3	28.1	10.2
	Between accessions within regions	295	139,757.9	473.7	226.7	82.4
	Within rice accessions	307	6247	20.3	20.3	7.4
	Total	613	166,765.3	272.0	275.2	100
Countries	Between countries	4	17,606.38	4401.60	43.30	14.87
	Between accessions within countries	412	196,919.92	477.96	230.10	79.03
	Within rice accessions	417	7402.66	17.75	17.75	6.10
	Total	833	221,928.96	266.42	291.15	100

### Comparison of Philippine pigmented Rice accessions with pigmented accessions from Neighbouring countries

**Fig. 6:**
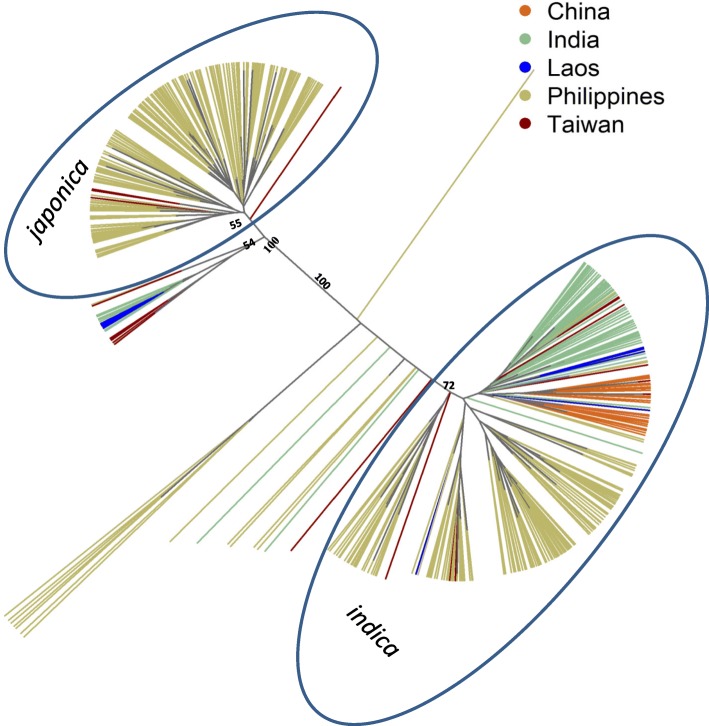
Unrooted neighbour-joining tree of 307 unique Philippine pigmented rice accessions and pigmented accessions from neighbouring countries. Numbers show bootstrap values for main branches

We compared the Philippine pigmented rice accessions with pigmented rice accessions from the neighbouring countries China, Taiwan, Laos and India to determine to what extent cross border movement of these Philippine rice accessions had occurred. There was little differentiation between Philippine pigmented rice accessions and pigmented accessions from Taiwan (*F*_ST_ = 0.04) and Laos (*F*_ST_ = 0.08). However, when compared with pigmented rice accessions from India (*F*_ST_ = 0.16) and China (*F*_ST_ = 0.23) the accessions were quite distinct. The average *F*_ST_ value among these countries was 0.14, indicating that 14% of the genetic diversity can be explained by population sub-structuring (Additional file 2: Table S2**)**. AMOVA indicated that the majority of genetic variation (79.03%) occurred within country populations, with 14.87% of the variation being between populations, and only 6.10% of genetic variation being within rice accessions, representing residual heterozygosity (Table 2). The highest genetic connection was seen between pigmented rice accessions from Taiwan and the Philippines, as indicated by the low level of genetic differentiation (*F*_ST_ = 0.04) and the close phylogenetic relationship between the majority of the accessions from Taiwan with accessions from the Philippines (Fig. 6).

### Selection of a genetic marker set for Rice accession identification

A set of 20 SNP markers that distinguished between individual genotypes within the 307 Philippine pigmented rice accessions was identified **(**Additional file 3: Table S12). To visualize the informativeness of this marker set, the number of polymorphic markers present in each pairwise comparison of the accessions was calculated (Additional file 1: Figure S10). Since only homozygous genotypes were considered in this analysis, a few highly heterozygous accessions had zero or very few polymorphic markers with other accessions. However, these heterozygous accessions could still be identified as distinct, having a identity-by-state genetic distance greater than 0.05 when compared to all other accessions.

### Screening for the 14-bp deletion within the Rice *Rc* locus known to result in loss of red pigmentation in Rice grains

A total of 241 genotypes ((192 red, 23 variable purple, 16 purple, 5 white, 4 mix (red and white) and 1 mix (red and variable purple)) from the 307 Philippine pigmented rice accessions were screened for the presence of the 14-bp deletion in the *Rc* gene (Additional file 1: Figure S11; Additional file 3: Table S13**)**. This deletion is known to result in the loss of red pericarp (Sweeney et al., 2006). This screen revealed that the functional allele of the *Rc* gene (red pigmented pericarp) was preponderant among the accessions screened. Of the 202 accessions for which a deletion was not observed, 84.16%, 9.41%, and 3.96% of the samples were reported as red, variable purple and purple, respectively, 1.49% of the samples had mixed colour, while 0.99% were white. A total of 29 rice accessions carried the *Rc* 14-bp deletion but still had pigmented pericarps ((15 red, 12 purple and variable purple accessions, and two mixed (red and white) accessions)), while two white (*japonica*) rice accessions did not carry the 14-bp deletion within the *Rc* locus. We recorded eight accessions, all with red pericarps, with ambiguous profiles, i.e. with more than one distinct band.

### Multi-spectral imaging of the Philippine pigmented rice accessions

A total of 10 rice seed traits were quantified; geometric traits - seed area, length, width and roundness, and seed colour parameters - lightness (L*), intensity, hue angle, saturation, a* (green – red; redness) and b* (blue – yellow; yellowness). The Shapiro-Wilk test indicated departure from a normal distribution (*P* < 0.05) for roundness, a*, b*, hue angle, intensity, saturation and lightness, while seed area, length and width showed no significant deviation from normality (*P* > 0.05) (Additional file 2: Table S5). Variability between rice accessions was observed for all traits (Additional file 1: Figures S12 & S13**)**. Hue angle was the less dispersed (CV = 0.07) trait, while saturation showed the highest dispersion (CV = 0.43). The highest lightness, a* and intensity values were reported in the red rice accessions, while the lowest lightness, a* and intensity were reported in the purple rice accessions. The lowest saturation and b* values were detected within variable purple accessions, while the highest values were found in a red accession (Additional file 2: Table S5).

Pearson’s correlation revealed several traits with significant correlations (Additional file 1: Figure S14). Significant correlations were observed between saturation and lightness, b* and intensity (r > 0.9). The highest positive correlation was observed between intensity and lightness (r = 0.99). Colour parameters a* and b* were highly correlated (r = 0.87). Negative and significant correlations were observed between roundness and width (r = − 0.70), and between hue angle and a* (r = − 0.62). Most geometric-related traits exhibited weak correlation with colour parameters.

**Fig. 7:**
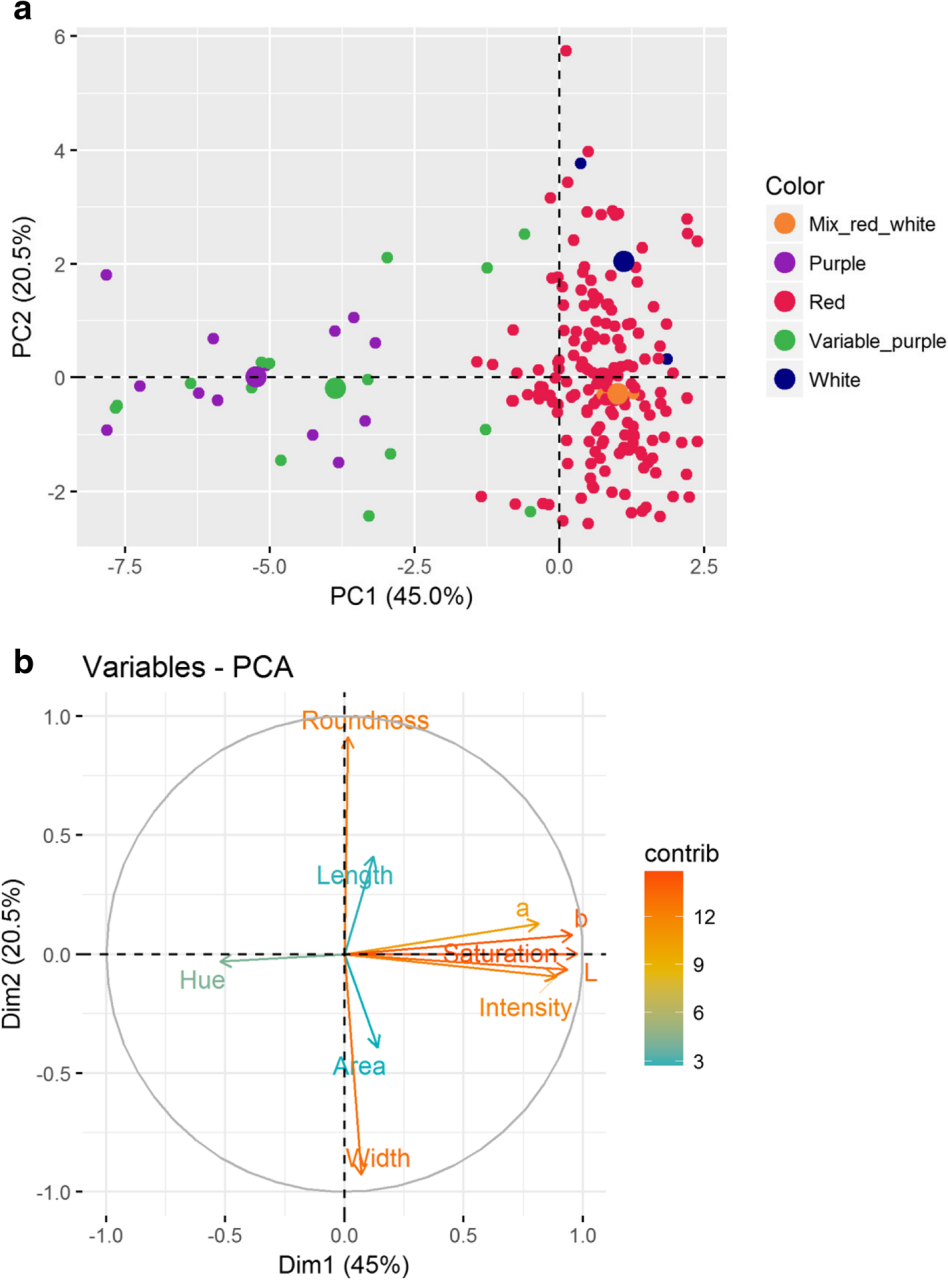
Principal component analysis of the 197 Philippine pigmented rice accessions used for multi-spectral imaging. **a** PCA showing the distribution of 197 rice accessions relative to visual colour assessment. **b** PCA trait distribution and contribution to the total variance

Principal component analysis of the geometric and colour-related traits separated the accessions into two main groups. The first group included purple and variable purple accessions, while the second group contained accessions with red, white and mix (red and white) seed colour. A few discrepancies were noted; four accessions reported as variable purple on the basis of visual classification now clustered closer to red accessions. The first principal components (PC) explained 45.0% of the phenotypic variation, while PC2 explained 20.5% (Fig. 7a). The variation in PC1 was associated with colour-related traits, while variation in the PC2 was driven by geometric-related traits (Fig. 7b).

An ANOVA revealed no significant associations between pericarp colouration and seed length (F_4, 192_ = 0.568, *p* = 0.686), area (F_4, 192_ = 0.827, *p* = 0.509), roundness (F_4, 192_ = 1254, *p* = 0.29) or width (F_4, 192_ = 0.851, *p* = 0.495), substantiating the weak correlations reported between geometric and colour traits. ANOVA analysis, followed by post-hoc comparisons using the Tukey HSD test, indicated a difference in colour intensity (F_4, 192_ = 74.96, *p* = 2e-16), saturation (F_4, 192_ = 143.7, p = 2e-16) and lightness (F_4, 192_ = 109.2, *p* = 2e-16) between rice accessions with different pigmentation (Additional file 2: Table S6). Non-homogeneity in means was observed for colour parameters a*, b* and hue angle, indicating the diverseness of the accessions for these traits. Heterogeneity also indicates that pericarp colouration could have differential effects on these particular traits across the accessions.

## Discussion

The Philippine traditional pigmented rice accessions held at the PhilRice Genebank represents a valuable collection of rice accessions for assessment of rice nutritionals. Previous, large-scale studies of pigmented rice accessions have used only morphological characteristics to assess diversity (Rabara et al. 2014), while molecular diversity studies have involved limited numbers of pigmented rice accessions (Patel et al. 2014; Ahmad et al. 2015; Ashraf et al. 2016). This study represents the first extensive genetic assessment of a large collection of pigmented rice accessions, using state-of-the-art SNP markers. The study demonstrates the continued exchange of these rice accessions between regions and the importance of pigmented rice to ethnic communities.

Almost half of the pigmented rice accessions retrieved from the PhilRice Genebank represented near-identical genotypes, a common phenomenon among genebank collections (Moura et al. 2013; Virk et al. 1995; Zhou et al. 2015). Multiple analytical approaches consistently divided the accessions into two major groups, *indica* and *japonica*, with considerable genetic differentiation (*F*_ST_ = 0.50) reflecting the distinctiveness and barriers to crossing that normally prevents gene flow between these two sub-species (Ouyang 2013). A few admix accessions were observed, although several of these accessions displayed a high degree of heterozygosity, unusual for a typically inbreeding species like rice. Therefore, it cannot be ruled out that these admix accessions are artifacts, caused by outcrossing prior to collection or during seed increase. Further work would be needed to unravel the true source of admixture and high heterozygosity in these rice accessions.

The genetic diversity within regions was moderate, but did vary across regions. The highest within region genetic diversity was reported in CAR and Mindanao 3 (He = 0.34), while the rice accessions from NCR showed the least diversity (He = 0.26). Genetic diversity is considered a function of a population’s ecological and evolutionary history (Choudhury et al. 2013). The observed genetic diversity could be associated with the diverse agro-climatic, eco-geographical, as well as ethno-cultural features of the regions. The varied eco-geographic features of CAR, including different altitudes, different climatic environments and limited accessibility, might contribute to the maintenance of landraces diversity (Sajise et al. 2012). CAR is also composed of diverse ethno-linguistic groups and Wang et al. (2016) have highlighted the positive influence of ethnic diversity on rice landrace genetic diversity. The southern–central region of Mindanao 2 also displayed substantial within region genetic diversity (He = 0.32). This landlocked area is home to a considerable number of rice varieties, being the traditional “rice bowl” of the Philippines (Tadem 2012).

Although *japonica* rice accessions were the most abundant subspecies within the Philippine pigmented rice accessions (*japonica* - 53.42%; *indica* – 43%), *indica* types predominated in CAR (48.9%). *Indica* rice is normally grown in low altitude and latitude areas, while *japonica* rice is favored in mountainous regions (Lu et al. 2009). As CAR is a mountainous area we would have expected a preponderance of *japonica* accessions, not *indica*. Our results support the findings of Cui et al. (2017), that *indica* types adapt to a wider range of conditions than *japonica* types, suggesting that *indica* accessions adapted better to the high altitude zones found in CAR. A significant number of *japonica* accessions were also collected in lowland regions, showing that the *japonica* accessions were tolerant to warmer climatic conditions. These accessions may be a valuable asset for breeding climate-resilient rice varieties.

The high percentage of molecular variance observed within regions (82.38%) is indicative of regular seed movement between local farmers. We would also expect farmers to maintain genetic diversity, choosing to grow genetically diverse varieties. However, low genetic differentiation between rice accessions grouped by region is evidence of seed exchange between regions. Long distance transfer of rice seed was apparent between the regions of Calabarzon and Mindanao 1, Mindanao 2 and Mindanao 3, and between MIMAROPA and Mindanaol 1. Rice seed movement was also apparent between Bicol and the regions of Central, Eastern and Western Visayas. In addition there was evidence for movement of rice accessions between the regions of Ilocos and NCR. Barter trading for goods is still frequent among rural farmers. Likewise, social and cultural practices, including giving rice as a gift, offerings as part of religious and spiritual practices, and during community events such as festivities could all have contributed substantially to movement of rice seeds (Sajise et al. 2012; Vilayheuang et al. 2016).

The close genetic connection between pigmented rice accessions from the Philippines with those from Taiwan supports the hypothesis of Southward diffusion of Austronesians from Taiwan to the Philippine (Bellwood 2011; Ko et al. 2014; Mörseburg et al. 2016). First domesticated in the Yangtze valley in China (Molina et al. 2011), *japonica* rice cultivation rapidly spread throughout Southeast Asia, mainly driven by demographic expansion (Bellwood 2011). Archeological evidence has shown that early Austronesians migrated from the Yangtze valley to the Fujian basin. As the regions became less conducive to farming after post-glacial warming, they migrated to Taiwan in search of better agricultural environments (Bellwood 2011, 2017; Ko et al. 2014). Population growth in Neolithic Taiwan and landscape degradation is believed to have caused human displacement from Taiwan to the northern Philippines, carrying their rice landraces with them (Bellwood 2013; Halili 2004).

Screening of the *Rc* locus confirmed the role of the 14-bp deletion in exon 6 (*rc* allele) in rice pericarp pigmentation (Sweeney et al. 2006, 2007). The majority of accessions without the 14-bp deletion possessed red pericarps, while most accessions carrying the *rc* allele had a white pericarp. However, not all white pigmented accessions carried the 14-bp deletion, suggesting that allelic variation elsewhere in the *Rc* locus may have resulted in loss of pigmentation. More interesting was the discovery of 29 pigmented rice accessions that did carry the *Rc* 14-bp deletion, but had not resulted in loss of pericarp pigmentation. These results would indicate the presence of other mutations, either in *Rc* (regain of function mutation) or other genes, that effect pericarp pigmentation (Gross et al. 2013).

Other mutations in exon 6 of the *Rc* gene have been identified that result in loss of red pigmentation. These include a C to A transversion found in an *aus* rice background (*Rc-S* allele; Singh et al. 2017; Sweeney et al. 2007) and a further point mutation, giving alleles *rc-gl,* found in an African rice (Gross et al. 2013). Brooks et al. (2008) identified a natural mutation (allele *Rc-g*) in *rc* that reverted white rice pericarp to red. The *Rc-g* mutation was caused by a 1-bp deletion, 20 bps upstream of the *rc* 14-bp deletion in exon 6, that restores the reading frame to a functional allele. Considerable genetic variation has been found in the *Rc* gene, with Singh et al. (2017) reporting 4 haplotypes (*Rc*-H1, *Rc*-H2, *Rc*-H3, *Rc*-H4), Cui et al. (2016) reporting high haplotype diversity in Malaysian rice, and Sun et al. (2018) reporting several functional and non-functional haplotype combinations in natural rice germplasm.

Purple or black rice is the result of the accumulation of anthocyanin in the pericarp, while the accumulation of proanthocyanidins results in red pigmentation. However, it is still unclear how regulation of many of the genes in the anthocyanin pathway affects the profile and quantity of flavonoids, and thus the intensity of colour in pigmented rice. Previous studies have highlighted a number of genes required for anthocyanin pigmentation in rice (Sakamoto et al. 2001, Sun et al. 2018) and more specifically in rice pericarps (Furukawa et al. 2006; Sweeney et al. 2006; Caixia and Qingyao 2007; Maeda et al. 2014; Oikawa et al. 2015; Rahman et al. 2013, 2016). Screening the core collection with these genes would be a valuable approach to begin to understand the genetics underlying pigmentation in the Philippine pigmented rice accessions.

Various studies have reported that the lightness (L*), redness (a*) and yellowness (b*) values of pigmented rice are strong indicators of bioactive components (Pramai and Jiamyangyuen 2016; Sedghi et al. 2012; Shao et al. 2018). As found in this study, Murdifin et al. (2015) reported that out of thirteen Indonesian pigmented rice genotypes the darkest grain showed the lowest L* value. A correlation between L* values and anthocyanin and phenolic content, as well as antioxidant activity meant that low L* values could be used as an indicator of high anthocyanin content and antioxidant activity (Yodmanee et al. 2011; Murdifin et al. 2015). Therefore, multi-spectral imaging colour parameters can provide insights into key nutritional characteristics, serving as a proxy to identify beneficial rice genotypes with high nutritional potential.

Some disagreement between visual colour assessment and multi-spectral imaging was observed. In total 3.04% of the rice accessions analysed by multi-spectral imaging were misplaced in cluster analysis. Four rice accessions with variable purple pericarps and two with white pericarps clustered with red accessions, highlighting the challenges associated with visual colour assessment. Purple and variable purple accessions did not show a clear separation, highlighting the challenges associated with image processing of bi-colour samples, compared to mono-coloured accessions, because of colour transition areas (Unay et al. 2010). Principal component analysis clearly discriminated purple accessions from accessions with red, mixed (red and white) and white pericarps, however, it did not separate red accessions from white accessions as previously reported (Shao et al. 2018).

## Conclusion

A genetic analysis of 307 unique pigmented rice accessions, gathered from across the Philippines, indicated that these accessions had been subject to both local and long-distance exchange, the majority of the genetic variation being within regions rather than between regions. Displaying pigmentation in the pericarp ranging from purple, red to brown, these rice accessions represent a valuable commodity to local cultures. However, efforts to develop high yielding pigmented rice varieties have been less than optimal. A study of Sri Lankan varieties indicated that new, higher yielding brown rice varieties contained considerably less beneficial nutritionals than old, traditional Sri Lankan rice landraces (Gunaratne et al. 2013). The data generated on this core collection provides a valuable resource by which to further assess the breeding potential of these accessions. The set of 20 SNP markers will support identification of the rice accessions, enable certification and justify the premium prices that are currently demanded for Philippine heirloom rice. Using modern SNP assay technologies the 20 SNP minimal fingerprints should cost less than $2 per sample. This core collection also provides a valuable research platform from which the genetics and biochemistry underlying pigmentation in rice pericarps can be analysed.

## Methods

### Plant material, DNA extraction and fingerprinting

Pigmented rice accessions, collected from all administrative regions of the Philippines, were provided by the Genebank at PhilRice (Fig. 5, Additional file 1: Figure S15). A total of 696 accessions were collected from 63 provinces across the Philippines (Fig. 5). Accession passport data was recorded when available (Additional file 2: Table S7). Visual assessment of the seed colour of the original 696 rice accessions was undertaken using Biodiversity indicators for pericarp colour (Bioversity International et al. 2007). DNA was extracted from a single seedling leaf of each rice accession using the oKtopure™ robotic DNA extraction system (http://www.lgcgroup.com) (Thomson 2014). Genotyping of 610 rice accessions was performed using the Illumina Infinium 6 K rice SNP chip (Thomson et al. 2017). Subsequent genotyping of the remaining 86 rice accessions used the updated 7 K Illumina Infinium SNP chip (http://gsl.irri.org/genotyping/infinium-7k, unpublished). Twenty pigmented rice accessions from Taiwan, including 17 red and 3 purple accessions, were retrieved from the IRRI Genebank and genotyped using the Illumina Infinium 7 K SNP chip (http://gsl.irri.org/genotyping/infinium-7k, unpublished) (Additional file 2: Table S8). A total of 4551 markers, common between the 6 k and 7 k Illumina arrays, were subjected to quality control analysis. The SNP data sets from the 6 k and 7 k Illumina array screens has been deposited on the Genotyping Service Laboratory, IRRI website (http://gsl.irri.org/resources/downloads/colored-rice-paper). Genotypic data of red pigmented accessions from China, India and Laos were extracted from the Rice SNP-Seek Database (http://snp-seek.irri.org; Mansueto et al. 2017). SNPs in common between the Rice SNP-Seek dataset and the 7 K SNP chip (http://gsl.irri.org/genotyping/infinium-7k, unpublished) were retrieved.

### Quality control of SNP data and development of a Core collection

Control quality of the genotypic data was performed using SNP & Variation Suite (SVS v8.6.0) (Golden Helix, Inc., Bozeman, MT, USA). Markers with call rates lower than 0.95 (call rate < 0.95) or minor allele frequencies (MAF < 0.05) were removed from the data set of 4551 SNP markers. The remaining SNPs were screened for redundant markers using the software’s default parameters (Window size = 2921; Window increment = 5; LD threshold = 0.8 and LD computation method = CHM), resulting in a final, usable data set of 1536 evenly distributed markers.

Rice accessions with low genotype call rates (call rate < 0.95) were removed from further analysis, leaving 589 rice accessions. The relatedness between rice accessions was also calculated using SNP & Variation Suite. Where rice accessions had similar genotypic SNP marker profiles (i.e. estimated probability of identity = 1) one accession was removed from further analysis (282 accessions removed), leaving 307 distinct rice accessions subsequently referred to as the core collection (Additional file 2: Table S1).

### Assignment of pigmented Rice accessions into *Oryzae sativa* varietal groups based on genetic distance

In order to assign the 696 Philippine pigmented rice accessions to rice varietal groups a cluster analysis was undertaken with the 3027 rice accessions in the 3 k SNP-Seek database (Mansueto et al. 2017). A total of 4539 SNP markers present in both the 6/7 k Illumina datasets and the 3 k SNP-Seek database were used for this clustering analysis. A genetic distance matrix among all pairs of samples was computed using a modified Euclidean distance model and a Neighbor-joining algorithm in Tassel 5 (Bradbury et al. 2007). The genetic distance matrix was exported from Tassel 5 as a Darwin Dis file to build a distance-based dendogram using the software Darwin 6.0 (Perrier and Jacquemoud-Collet 2006). PCA was performed on the genetic distance matrix using the prcomp command of R statistical software (RStudio Team 2016). The varietal group of each pigmented rice accession was inferred based on the varietal group designation of close-by 3 k rice accessions.

### Assessment of the population structure within the Philippine pigmented Rice accessions

Maximum likelihood estimates, implemented in the ADMIXTURE program (Alexander and Lange 2011) was carried out on the 589 rice accessions that passed control quality (data not shown), and again on the 307 unique accessions of the core collection. This analysis was carried out using the CoARE facility of the DOST-Advanced Science and Technology Institute (DOST-ASTI) and the Computing and Archiving Research Environment (CoARE) project. A tenfold cross-validation procedure was used to define the most suitable K-value. The number of K-value computes ranged from 1 to 15. The proportion of the putative ancestral population of each rice accession was defined in the Q-matrix. Rice accessions were assigned to a group if the probability of their group membership, as determined by ADMIXTURE, was ≥80%. Accessions with less than 80% probability of a single group membership were classified as admix. Potential substructure within the main groups was investigated by carrying out structure analysis. The data set for each main group was independently analysed using the maximum likelihood approach implemented in the ADMIXTURE program (Alexander and Lange 2011).

The genetic distance between all possible pairs of rice accessions were calculated using the bitwise.dist function implemented in the R package Poppr (Kamvar et al. 2014). The pairwise genetic distance matrix generated was used to visualize relationships between accessions. Phylogeny trees were constructed and visualized using the neighbor joining method implemented in the R package ape (Paradis et al. 2004). Bootstrap analysis was performed using the aboot function implemented in the R package poppr (Kamvar et al. 2014), and branch supports estimated. The bootstrap support values were exported and viewed using Figtree v.1.4.3 (http://tree.bio.ed.ac.uk/software/figtree/).

The level of genetic affinity between rice accessions were also analyzed by principal component analysis (PCA) using the R package rrBLUP (Endelman 2011). PCA was performed using the prcomp command of R statistical software (RStudio Team 2016).

### Estimates of genetic diversity within the Philippine pigmented Rice accessions

The 1536 SNP markers were used to assess genetic diversity among the 307 accessions of the core collection. The following metrics were estimated by rice subspecies and region of origin; the number of alleles (Na), observed heterozygosity (Ho), expected heterozygosity (He), and the inbreeding coefficient (FIS). FIS significance was assessed using a 95% confidence interval and a 1000 bootstrap replicates estimate of genetic diversity. Estimates of genetic diversity were performed with the R package diveRsity using the divBasic function (Keenan et al. 2013). Expected heterozygosity, as well as inbreeding coefficients, were also estimated for each individual rice accession using Golden Helix SNP & Variation Suite (Golden Helix, Inc., Bozeman, MT, USA). Significance differences in diversity between groups was estimated using Wilcoxon signed rank test in R (RStudio Team 2016).

### Analyses of molecular variance (AMOVA) and population differentiation

Analysis of molecular variance (AMOVA) was performed with the aim of determining the partition of genetic variation among populations and/or groups. These analyses were carried out using the R package Poppr (Kamvar et al. 2014). Population differentiation was evaluated using pairwise *F*_ST_ estimate, according to Weir and Cockerham (1984) using Golden Helix SNP & Variation Suite (Golden Helix, Inc., Bozeman, MT, USA).

### Genetic marker set for individual Rice accession identification

To identify an effective set of genetic markers that could distinguish any accession within the given data set, we used a Java program *purity* (http://bitbucket.org/jcignacio/purity) that utilizes a genetic algorithm (Wilhelmstotter 2017) to maximize the number of uniquely identified genotypes. A HapMap file of 307 rice accessions and 1536 markers was used as input, and a subset of 20 SNP markers selected. The program was run with no mutation rate, a partially matched crossover rate of 0.35, a survival rate of 0.30 and a population size of 50,000. These parameters were selected after running multiple values for each parameter and selecting the values at which the data plateaued. An optimal solution that identified all 307 accessions was obtained after two iterations, with minimum 0.05 identity-by-state genetic distance between distinct samples. Out of the 20 SNPs, the number of polymorphic markers for all pairs of rice accessions was counted by considering only homozygous alleles.

### Relationship between Philippines pigmented Rice accessions and pigmented Rice accessions from Neighbouring countries

Genotypic data of pigmented rice accessions from neighbouring countries, including China, India, Laos and Taiwan, present in the 3 k SNP-Seek database (Mansueto et al. 2017) were combined with the SNP data for the 307 rice accessions from the Philippines. Genetic distances between samples were estimated and NJ tree constructed as aforementioned. The accessions were later grouped by country of origin and the partition of genetic variation at different levels was calculated using AMOVA in R package Poppr (Kamvar et al. 2014). Population differentiation among groups was evaluated using pairwise *F*_ST_ estimate, according to Weir and Cockerham (1984) in Golden Helix SNP & Variation Suite (Golden Helix, Inc., Bozeman, MT, USA).

### Screening of the Philippine pigmented Rice accessions for the allele present at the *Rc* gene locus

The rice gene *Rc* has been shown to confer red pigmentation in rice pericarps, with a 14-bp deletion resulting is loss of pigmentation (Sweeney et al. 2006). An indel marker designed by (Sweeney et al. 2006) was used to screen the *Rc* locus in 241 randomly selected accessions from the 307 unique Philippine pigmented rice accessions. The PCR consisted of 2 μl of 50 ng/μl of genomic DNA, 0.8 mM dNTPs, 0.05 U Taq DNA polymerase, 1× PCR buffer with magnesium, 0.5 μM reverse and forward primers, in a final volume of 10 μl. Amplification of genomic DNA was carried out in a thermal cycler machine (G-storm GS1) using the following PCR profile: An initial denaturation step at 94 °C for 5 min followed by 35 cycles of, a denaturation step at 94 °C for 1 min, annealing at 72 °C for 5 min, and extension at 72 °C for 2 min, ending with a final extension at 72 °C for 5 min. PCR products were separated in a 8% non-denaturing polyacrylamide gel ran at 100 V for 2.5 h. The gel was stained with SYBR Safe DNA gel stain for 10 min and visualized using an AlphaImager HP gel imaging device (Cell Biosciences, CA, USA). PCR products were scored as binary data, with the presence of the deletion being scored as 0 and the absence of the deletion as 1.

### Multi-spectral imaging of the Philippine pigmented Rice accessions

Seed of 197 rice accessions from the core collection were dehulled and used for multi-spectral phenotyping (Additional file 2: Table S1). All digital images were captured using the VideometerLab 4 (https://videometer.com), calibrated with respect to intensity and geometric parameters, followed by light set up. Randomly selected seeds, representing the darkest and the lightest rice accessions were used for the light set up. Twenty seed from each accession were placed evenly across a 90 mm Petri dish. The multi-spectral image of each individual seed is captured at 19 different wavelengths from 365 to 970 nm, each with a resolution of 2056 × 2056 pixels (Hansen et al., 2016). Colour difference metrics defined by the CIE (*Commission International d’Eclairage*) in 1976 and colour-appearance attributes; a* (green to red shade), b* (blue to yellow shade), L (lightness, clarity of the pericarp), intensity, saturation (the saturation of a colour describes its degree of purity in relation to neutral grey) and hue (angular specification of the colour perceived as red, yellow, blue or green) were extracted and quantified, as well as geometric traits; area, length, width and roundness (an estimate of how closely the shape of the seed resembles a circle).

The average values (average of twenty seed) for each accession were calculated and used in further analyses. Descriptive statistics for each trait ((minimum and maximum values, range, median, variance, coefficient of variation (CV), standard deviation (SD)) were obtained using the R package pastecs describe function (Grosjean et al. 2018). Boxplots, in combination with the Shapiro-Wilk test, and histograms were used to visualize the variation in the phenotypic data sets, and to check for normality of the traits. Correlations between traits and their level of significance were conducted in the R package Hmisc using the function rcorr (Harrell et al. 2017). Correlogram was generated using library (“corrplot”). Rice accessions were assigned to homogeneous groups by principal component analyses using the R package factorMineR (Lê et al. 2008). A one-way analysis of variance (ANOVA) was conducted to compare the effect of pericarp colouration on the traits, in white, red, mix (red and white), variable purple and purple pericarp groups. Post-hoc comparisons using Tukey HSD test were undertaken when significant differences were observed. Prior to ANOVA residuals were tested for normality and homogeneity using the Shapiro-Wilk test and Bartlett test, respectively. When homoscedasticity was violated, the variable was transformed and the residuals again inspected for normality and homogeneity. Non-homogeneity in means was observed for colour parameters a*, b* and hue angle, even after transformation, therefore these traits were precluded from further testing.

## Additional files

Additional file 1: **Figure S1.** The proportion of the 696 Philippine pigmented rice accessions that fell within each varietal group. **Figure S2.** Heat map of IBD PI estimates of the 589 Philippine pigmented rice accessions. **Figure S3.** Heat map of IBD PI estimates of all the 307 unique accessions retained for downstream analyses. **Figure S4.** Neighbour-joining tree showing the phylogenetic relationships between the 307 Philippine pigmented rice accessions. **Figure S5.** Principal component analysis of 307 selected Philippine pigmented rice accessions. **Figure S6.** Population structure within the *indica* rice accessions from the core Philippine pigmented rice collection. **Figure S7.** Population structure within *japonica* rice accessions of the core Philippine pigmented. **Figure S8.** Principal component analysis of 307 core Philippine pigmented rice accessions. **Figure S9.** The level of heterozygosity in individual rice accessions making-up the 307 core collection. **Figure S10.** Comparison of SNP markers between the 307 Philippine pigmented rice accessions. **Figure S11**. Number of Philippine pigmented rice accessions having the 14-bp deletion within the rice *Rc* gene known to result in loss of red pericarp. **Figure S12**. Boxplots of the 10 multi-spectral traits assessed in rice seed of 197 of the Philippine pigmented rice accessions. **Figure S13.** Histogram showing the distribution of geometrics and colour-related parameters in the 197 Philippine pigmented rice accessions screened using multi-spectral imaging. **Figure S14.** Correlations between the 10 multi-spectral traits assessed in rice seed of the 197 Philippine pigmented rice accessions. **Figure S15.** Rice sample size by region among the 696 Philippine pigmented rice accessions. (PPTX 3107 kb)

Additional file 2: **Table S1.** The 307 unique Philippine pigmented rice accessions used to assess genetic diversity. **Table S2.** Pairewise *F*_ST_ comparison between rice varietal types and regions among the 307 unique accessions. **Table S3.** Pairewise *F*_ST_ comparison between regions considering only the * indica* accessions among the 307 unique accessions. **Table S4.** Pairewise *F*_ST_ comparison between regions considering only the *japonica* accessions among the 307 unique accessions. **Table S5.** Descriptive statistics of colour parameters and geometric-related traits. **Table S6.** Data: Intensity by Colour. **Table S7.** Passport information on the 696 Philippine traditional pigmented rice accession collected. **Table S8.** Accessions from neigbouring countries used in this study. (XLSX 171 kb)

Additional file 3: **Table S9.** Philippine pigmented rice accessions allocated to redefined geographical regions. **Table S10.** Summary statistics of genetic diversity indicators across regions comparing the Philippine pigmented rice accessions that belong to the *indica* and *japonica* accessions. **Table S11.** Analysis of molecular variance of the Philippine pigmented rice accessions after removing the admix accessions. **Table S12.** Unique set of SNP markers that distinguishes individual accessions within the 307 core collection of Philippine pigmented rice accessions. **Table S13.** Summary of Philippine pigmented rice accessions carrying the 14-bp deletion within the rice *Rc* locus. (DOCX 43 kb)

## Abbreviations

a*: (green – red redness)
AMOVA: Analysis of Molecular Variance
ANOVA: One-way analysis of variance
ARMM: Autonomous Region of Muslim Mindanao
b*: (blue – yellow; yellowness)
bHLH: Basic Helix-Loop-Helix, MAF: Minor allele frequency
CAR: Cordillera Administrative Region
CoARE: Computing and Archiving Research Environment
CV: Coefficient of variation
DOST-ASTI: DOST-Advanced Science and Technology Institute
Estimated PI: Estimated probability of identity
FIS: Inbreeding coefficient
*F*_ST_: Degree of genetic differentiation
He: Expected heterozygosity
Ho: Observed heterozygosity
L^*^: lightness
MIMAROPA: Mindoro, Marinduque, Romblon and Palawan
Na: Number of alleles
NCR: National Capital Region
NJ: Neighbor joining
PCA: Principal component nalysis
SD: Standard deviation
SOCCSKSARGEN: South Cotabato, Cotabato City, North Cotabato, Sultan Kudarat, Sarangani and General Santos City
