# Supplementary material for: Exploring the genetic diversity within traditional Philippine pigmented Rice
Source: Rice (N Y). 2019 Apr 30;12:27. doi: 10.1186/s12284-019-0281-2 (PMC6491523; doi:10.1186/s12284-019-0281-2)
Supplement: Supplementary file 1 — Figure S1. The proportion of the 696 Philippine pigmented rice accessions that fell within each varietal group. Figure S2. Heat map of IBD PI estimates of the 589 Philippine pigmented rice accessions. Figure S3. Heat map of IBD PI estimates of all the 307 unique accessions retained for downstream analyses. Figure S4. Neighbour-joining tree showing the phylogenetic relationships between the 307 Philippine pigmented rice accessions. Figure S5. Principal component analysis of 307 selected Philippine pigmented rice accessions. Figure S6. Population structure within the indica rice accessions from the core Philippine pigmented rice collection. Figure S7. Population structure within japonica rice accessions of the core Philippine pigmented. Figure S8. Principal component analysis of 307 core Philippine pigmented rice accessions. Figure S9. The level of heterozygosity in individual rice accessions making-up the 307 core collection. Figure S10. Comparison of SNP markers between the 307 Philippine pigmented rice accessions. Figure S11. Number of Philippine pigmented rice accessions having the 14-bp deletion within the rice Rc gene known to result in loss of red pericarp. Figure S12. Boxplots of the 10 multi-spectral traits assessed in rice seed of 197 of the Philippine pigmented rice accessions. Figure S13. Histogram showing the distribution of geometrics and colour-related parameters in the 197 Philippine pigmented rice accessions screened using multi-spectral imaging. Figure S14. Correlations between the 10 multi-spectral traits assessed in rice seed of the 197 Philippine pigmented rice accessions. Figure S15. Rice sample size by region among the 696 Philippine pigmented rice accessions. (PPTX 3107 kb) [file 12284_2019_281_MOESM1_ESM.pptx]

## Slide 1
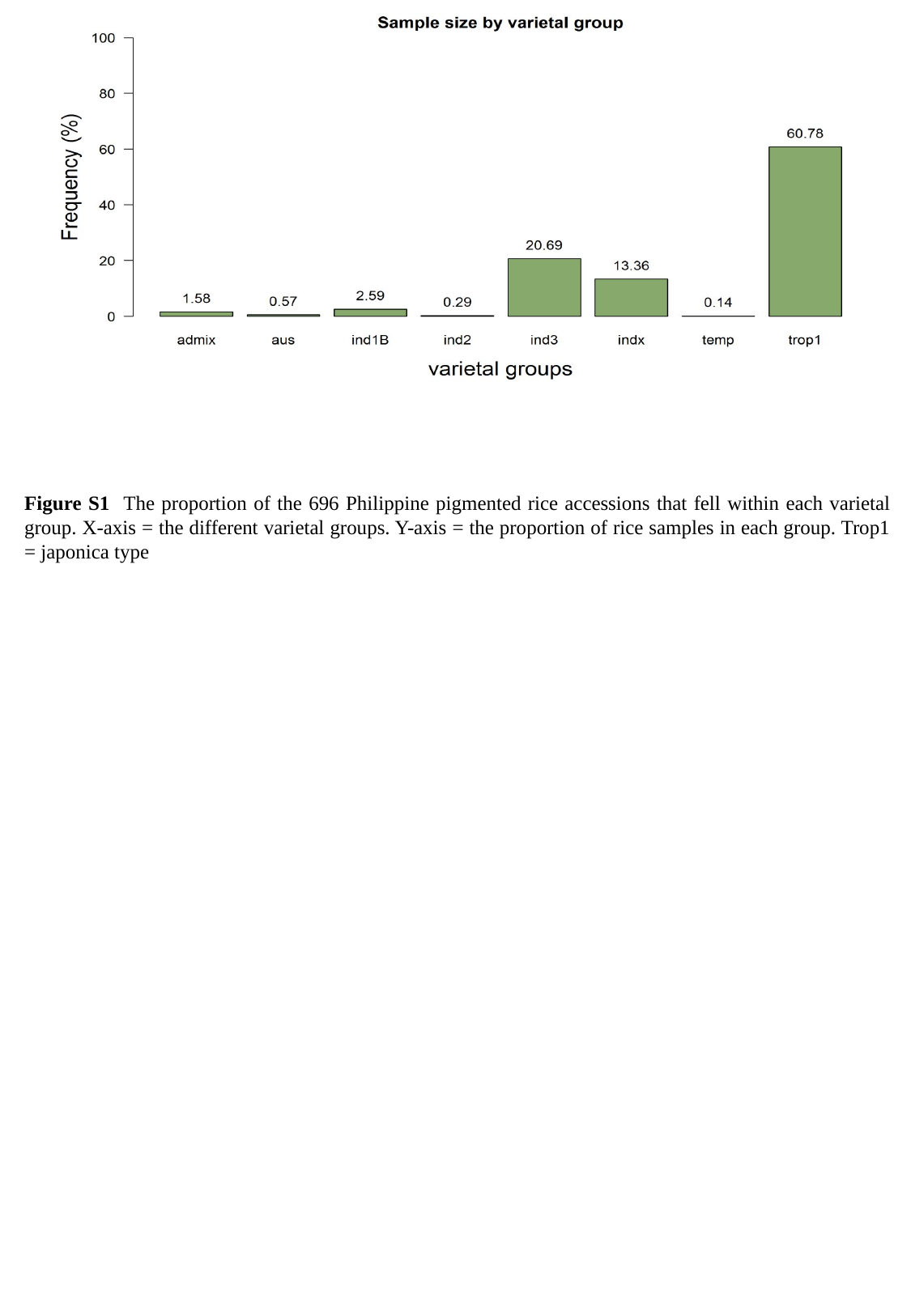

Figure S1 The proportion of the 696 Philippine pigmented rice accessions that fell within each varietal group. X-axis = the different varietal groups. Y-axis = the proportion of rice samples in each group. Trop1 = japonica type

## Slide 2
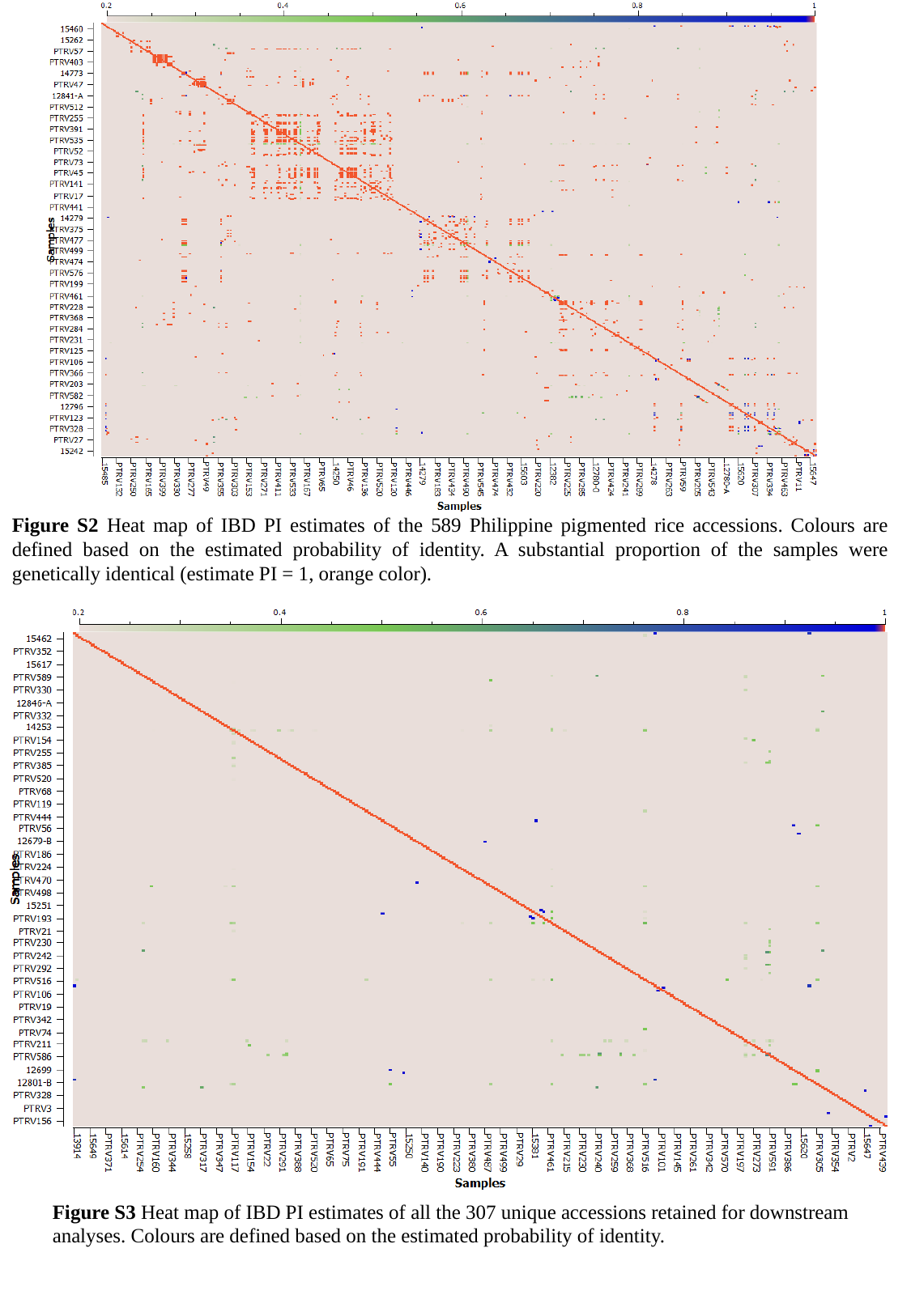

Figure S2 Heat map of IBD PI estimates of the 589 Philippine pigmented rice accessions. Colours are defined based on the estimated probability of identity. A substantial proportion of the samples were genetically identical (estimate PI = 1, orange color).
Figure S3 Heat map of IBD PI estimates of all the 307 unique accessions retained for downstream analyses. Colours are defined based on the estimated probability of identity.

## Slide 3
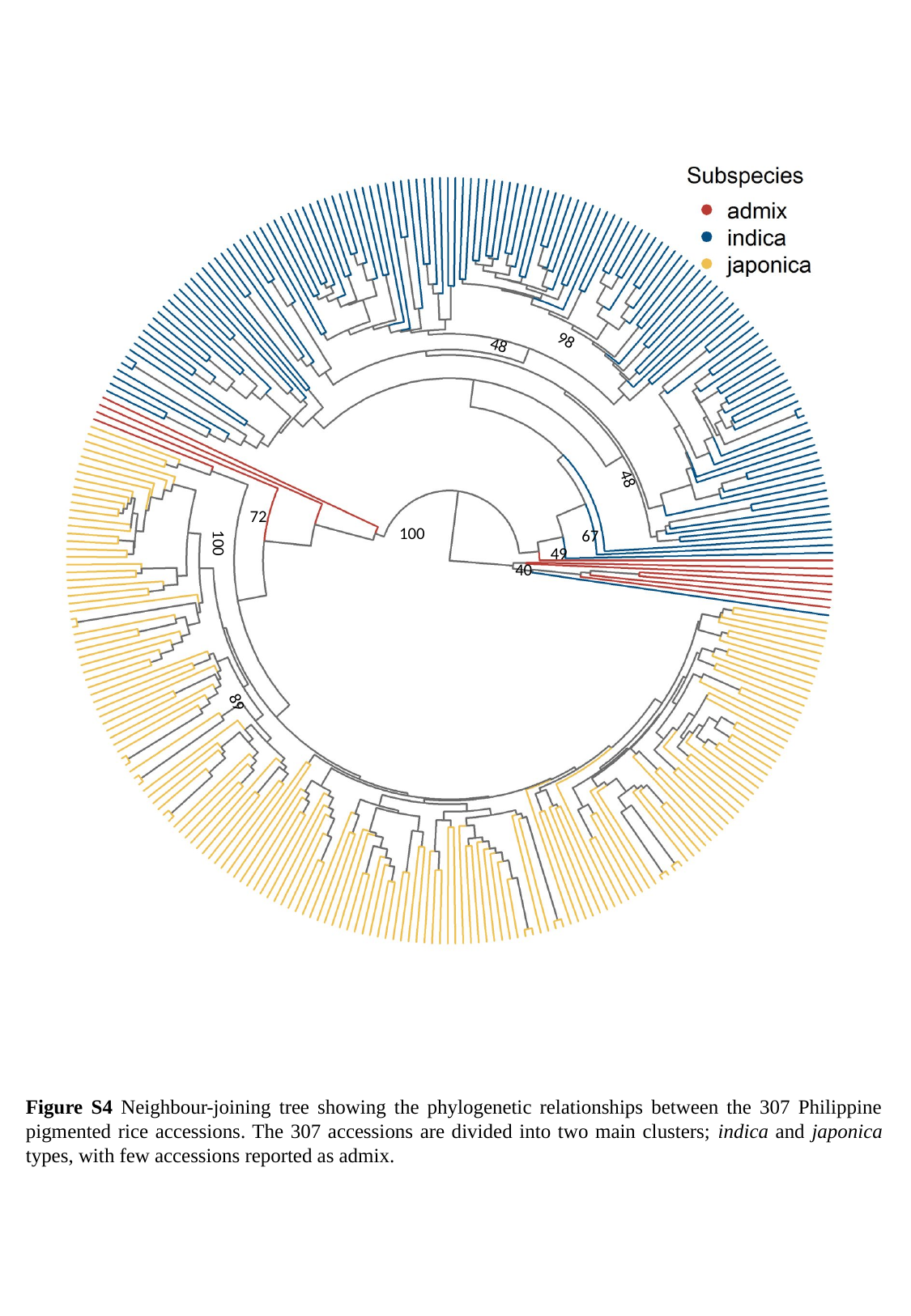

98
48
48
72
100
67
100
49
40
89
Figure S4 Neighbour-joining tree showing the phylogenetic relationships between the 307 Philippine pigmented rice accessions. The 307 accessions are divided into two main clusters; indica and japonica types, with few accessions reported as admix.

## Slide 4
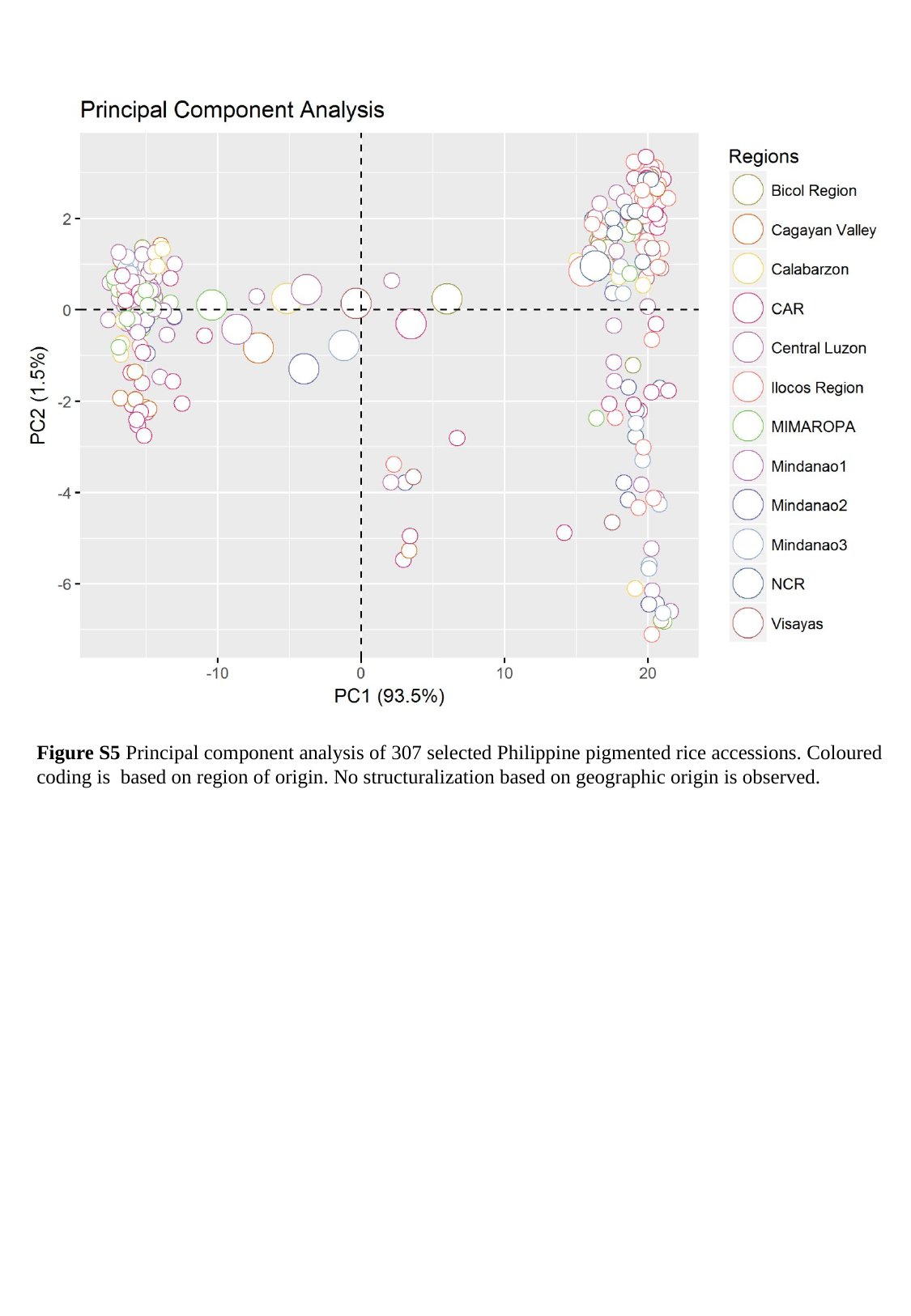

Figure S5 Principal component analysis of 307 selected Philippine pigmented rice accessions. Coloured coding is based on region of origin. No structuralization based on geographic origin is observed.

## Slide 5
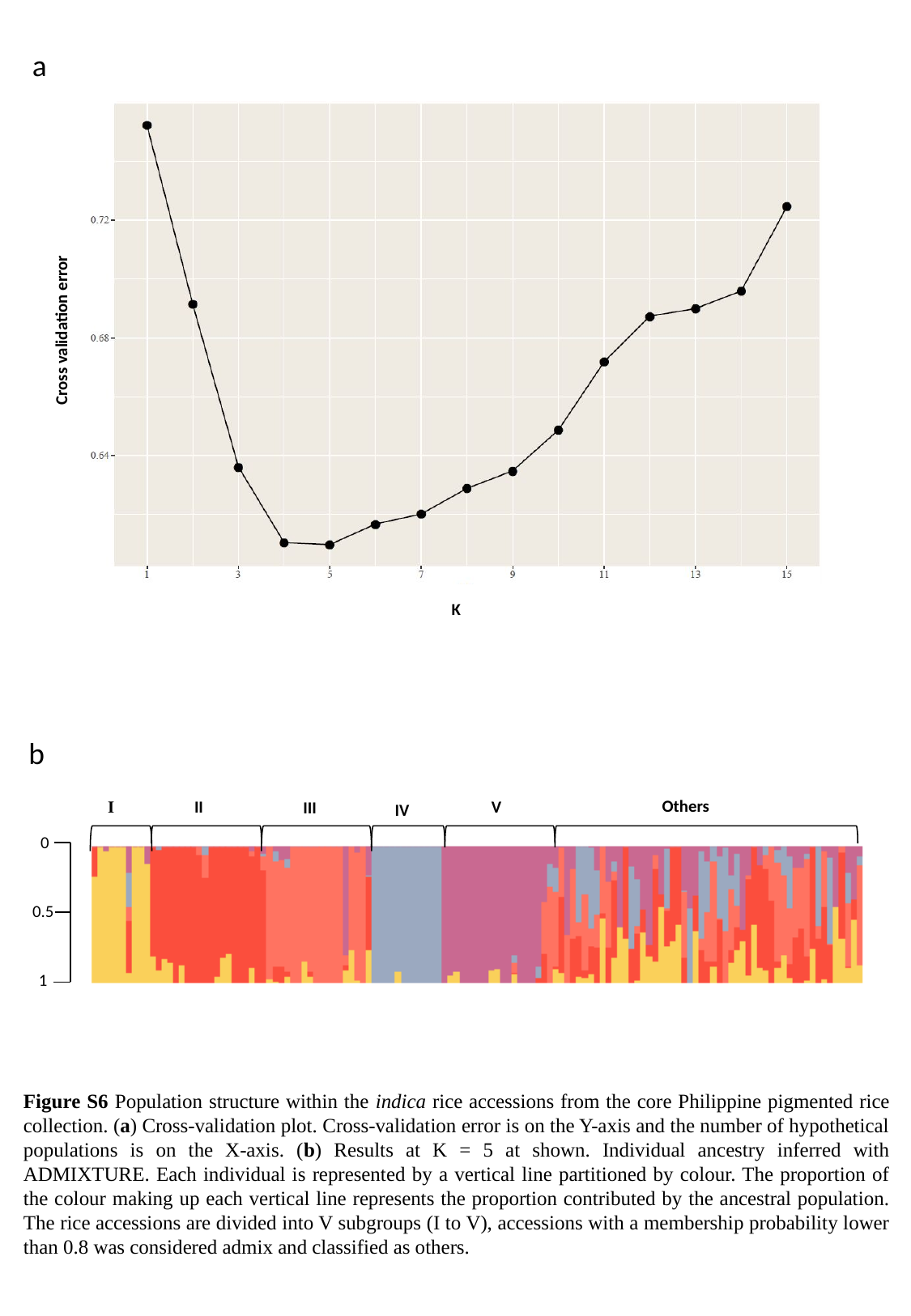

a
Cross validation error
K
b
Others
V
I
II
III
IV
0
0.5
1
Figure S6 Population structure within the indica rice accessions from the core Philippine pigmented rice collection. (a) Cross-validation plot. Cross-validation error is on the Y-axis and the number of hypothetical populations is on the X-axis. (b) Results at K = 5 at shown. Individual ancestry inferred with ADMIXTURE. Each individual is represented by a vertical line partitioned by colour. The proportion of the colour making up each vertical line represents the proportion contributed by the ancestral population. The rice accessions are divided into V subgroups (I to V), accessions with a membership probability lower than 0.8 was considered admix and classified as others.

## Slide 6
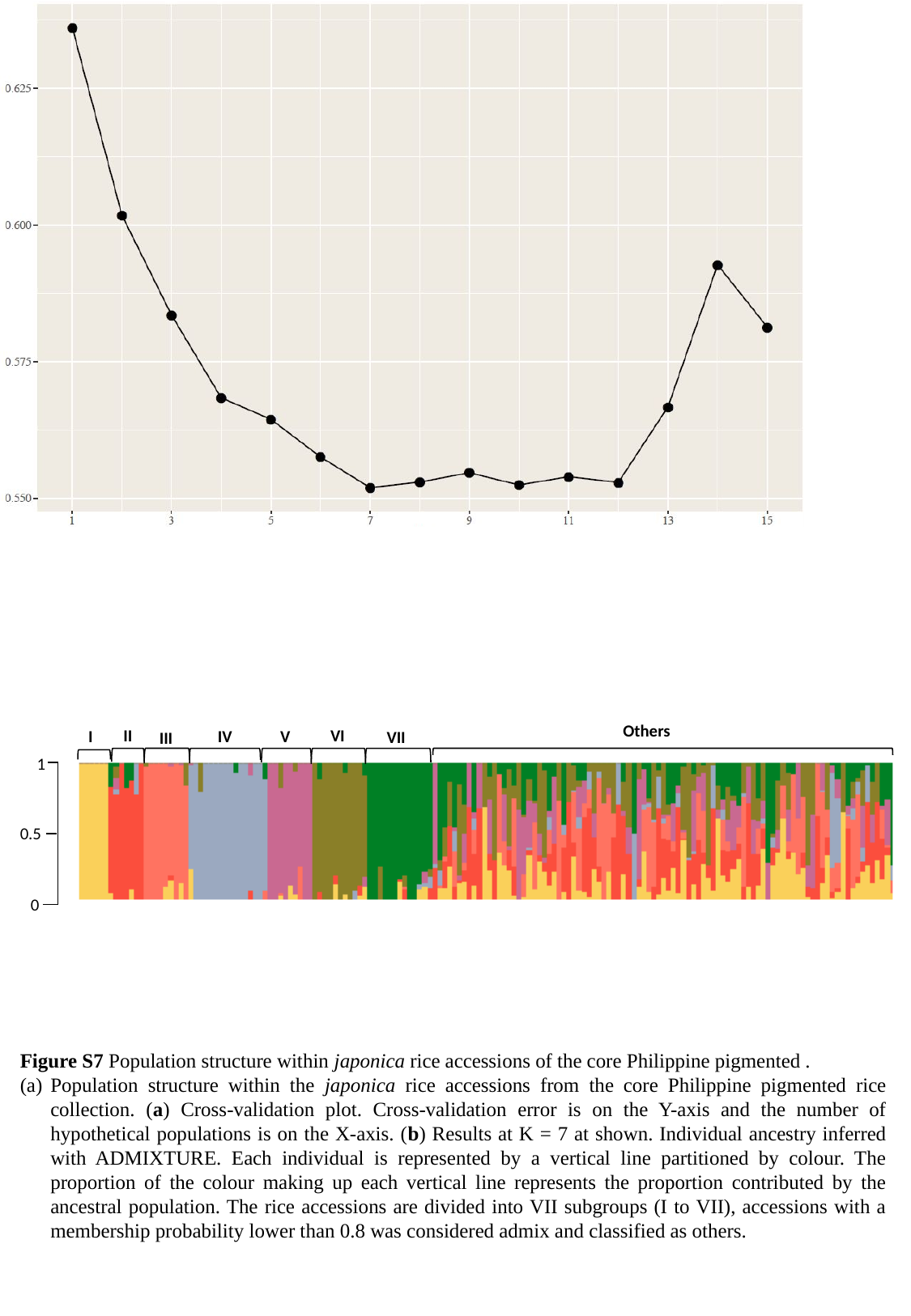

Others
VI
II
I
V
IV
VII
III
0.5
0
1
Figure S7 Population structure within japonica rice accessions of the core Philippine pigmented .
Population structure within the japonica rice accessions from the core Philippine pigmented rice collection. (a) Cross-validation plot. Cross-validation error is on the Y-axis and the number of hypothetical populations is on the X-axis. (b) Results at K = 7 at shown. Individual ancestry inferred with ADMIXTURE. Each individual is represented by a vertical line partitioned by colour. The proportion of the colour making up each vertical line represents the proportion contributed by the ancestral population. The rice accessions are divided into VII subgroups (I to VII), accessions with a membership probability lower than 0.8 was considered admix and classified as others.

## Slide 7
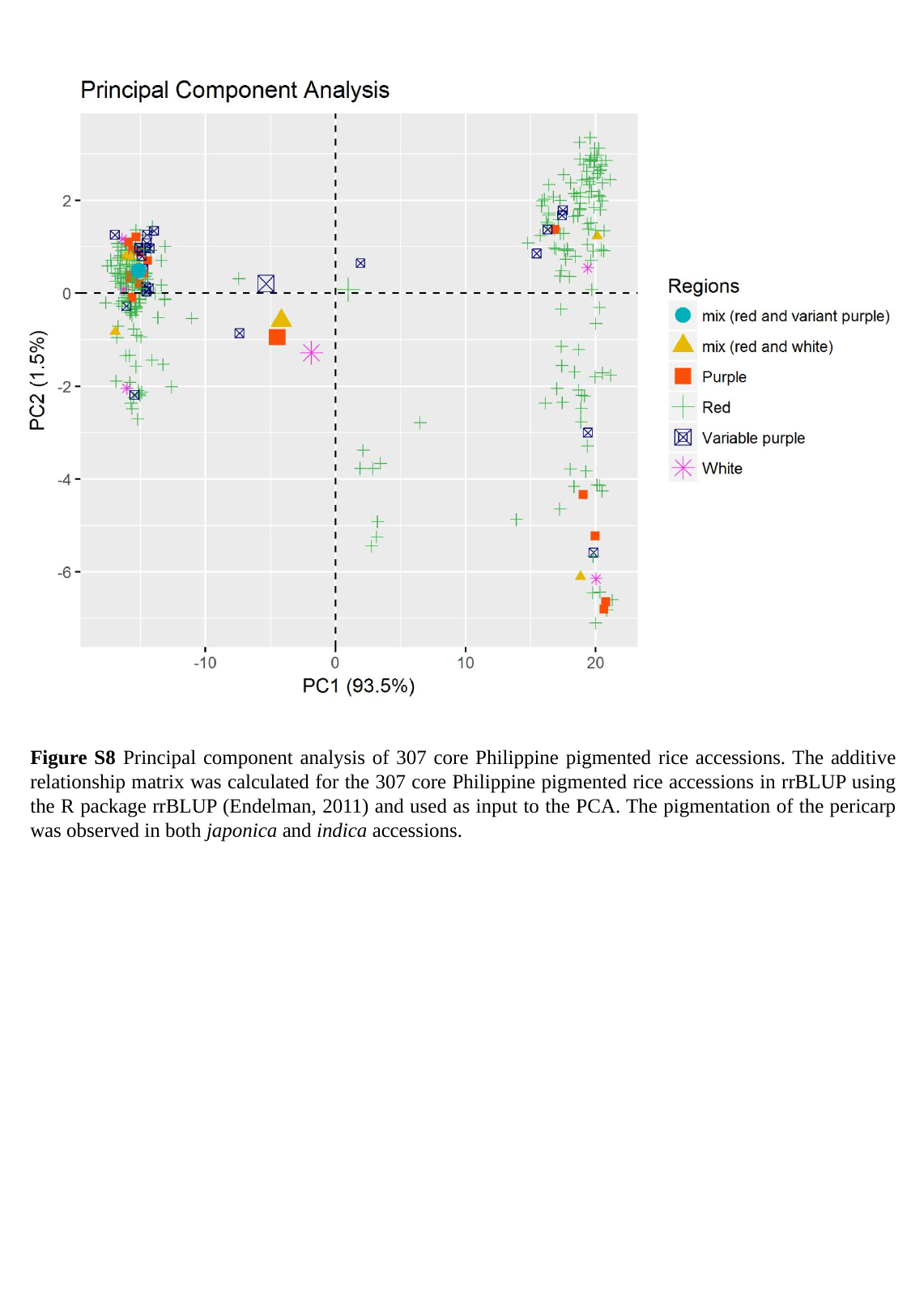

Figure S8 Principal component analysis of 307 core Philippine pigmented rice accessions. The additive relationship matrix was calculated for the 307 core Philippine pigmented rice accessions in rrBLUP using the R package rrBLUP (Endelman, 2011) and used as input to the PCA. The pigmentation of the pericarp was observed in both japonica and indica accessions.

## Slide 8
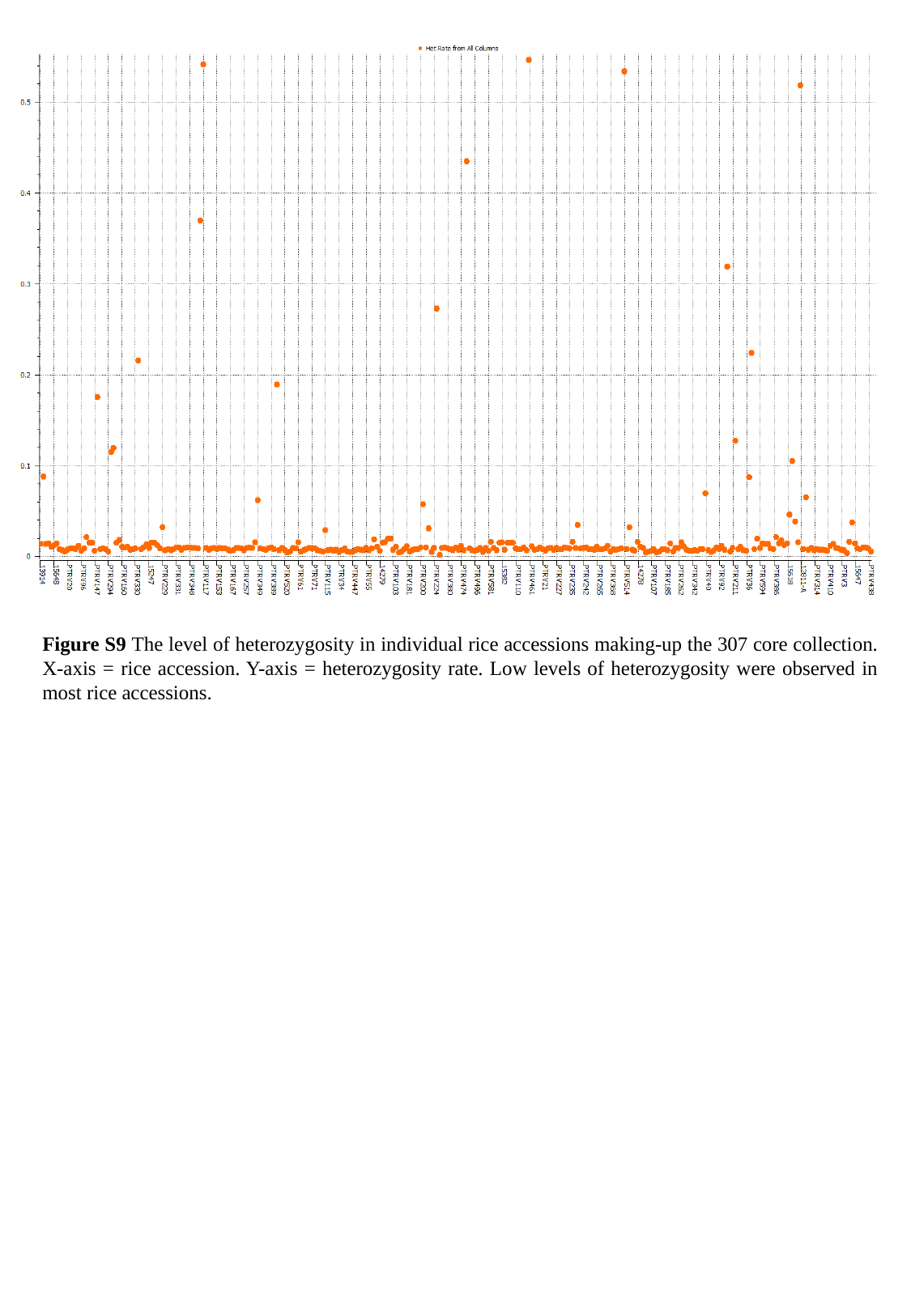

Figure S9 The level of heterozygosity in individual rice accessions making-up the 307 core collection. X-axis = rice accession. Y-axis = heterozygosity rate. Low levels of heterozygosity were observed in most rice accessions.

## Slide 9
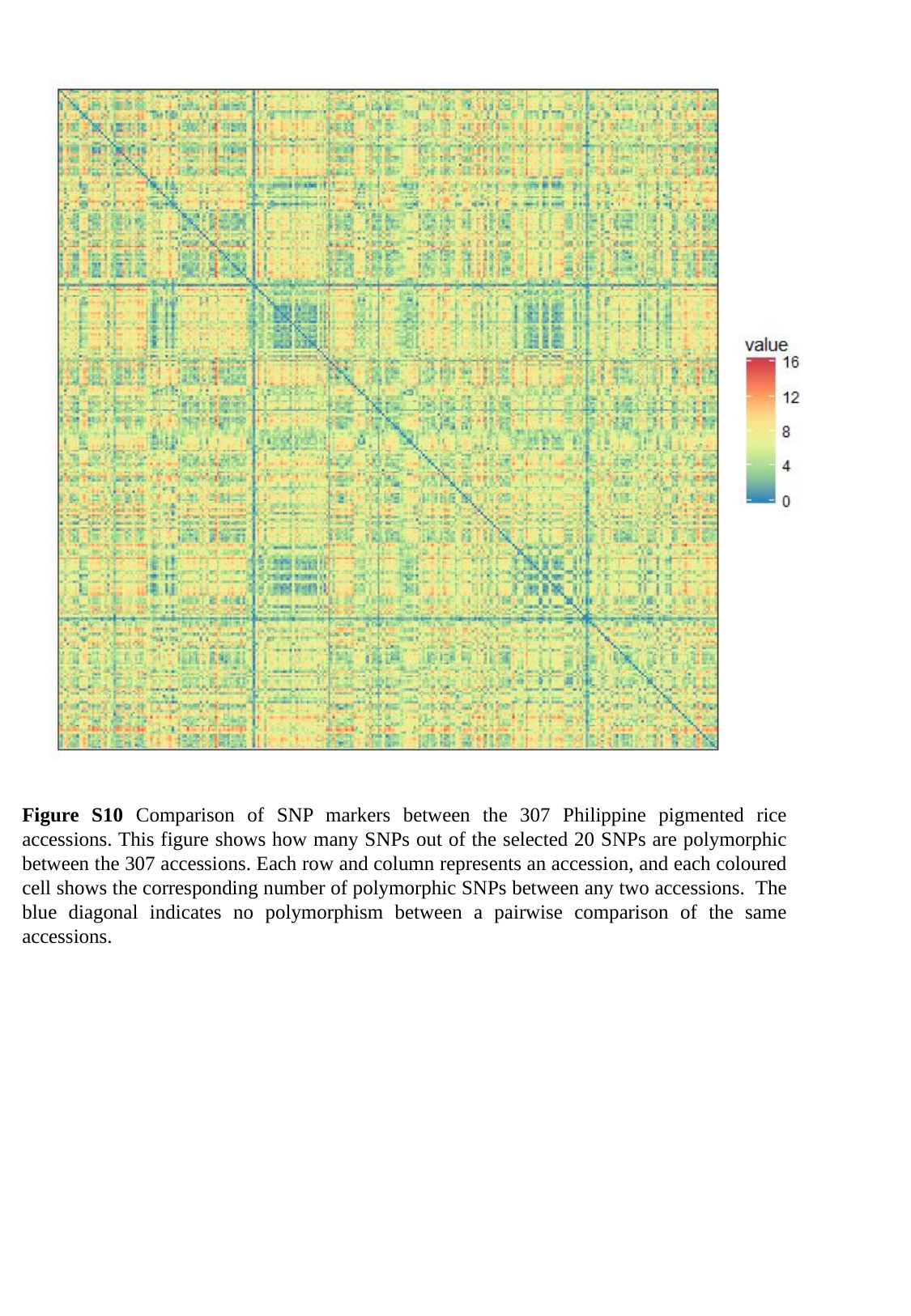

Figure S10 Comparison of SNP markers between the 307 Philippine pigmented rice accessions. This figure shows how many SNPs out of the selected 20 SNPs are polymorphic between the 307 accessions. Each row and column represents an accession, and each coloured cell shows the corresponding number of polymorphic SNPs between any two accessions. The blue diagonal indicates no polymorphism between a pairwise comparison of the same accessions.

## Slide 10
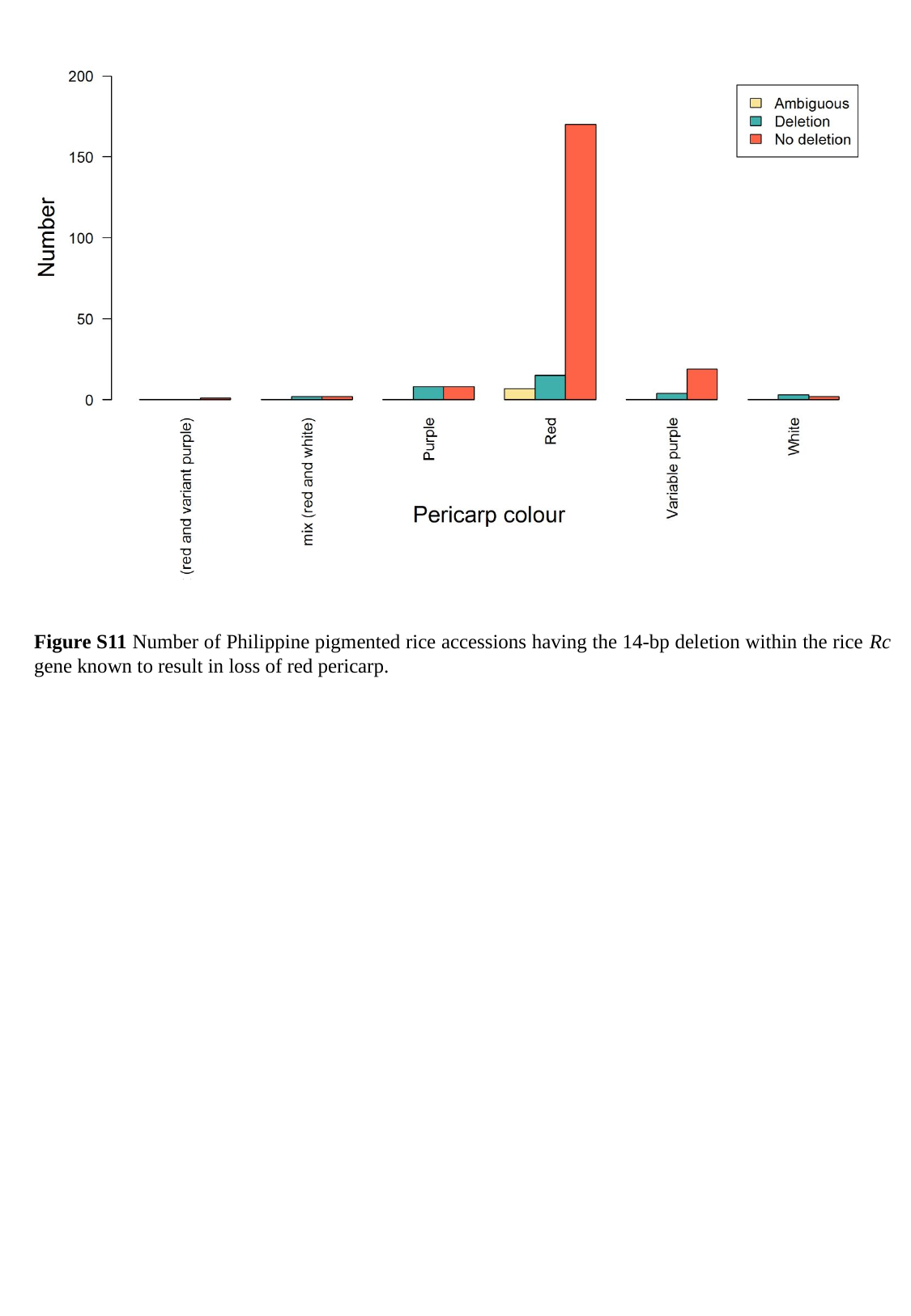

Figure S11 Number of Philippine pigmented rice accessions having the 14-bp deletion within the rice Rc gene known to result in loss of red pericarp.

## Slide 11
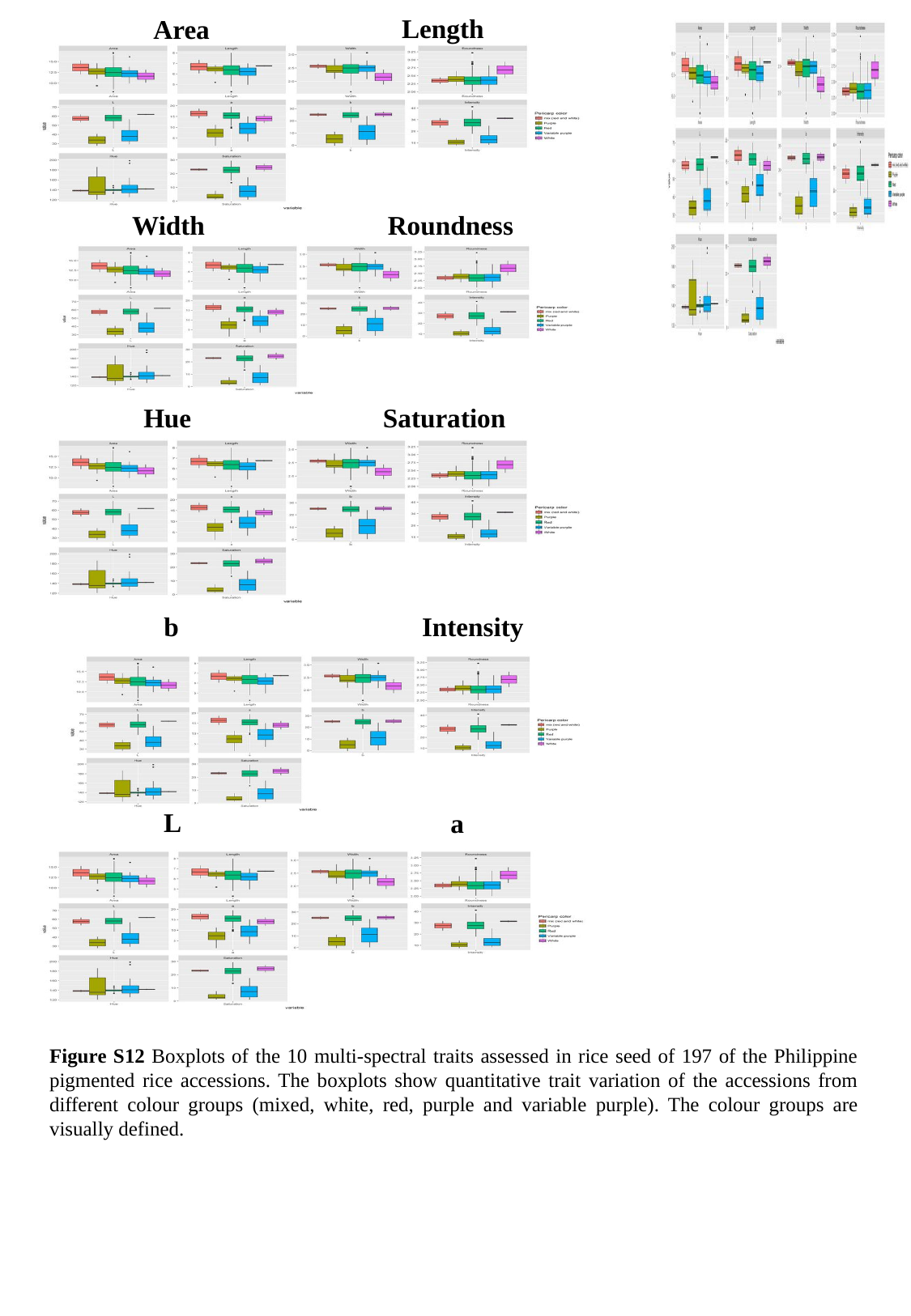

Length
Area
Width
Roundness
Hue
Saturation
b
Intensity
L
a
Figure S12 Boxplots of the 10 multi-spectral traits assessed in rice seed of 197 of the Philippine pigmented rice accessions. The boxplots show quantitative trait variation of the accessions from different colour groups (mixed, white, red, purple and variable purple). The colour groups are visually defined.

## Slide 12
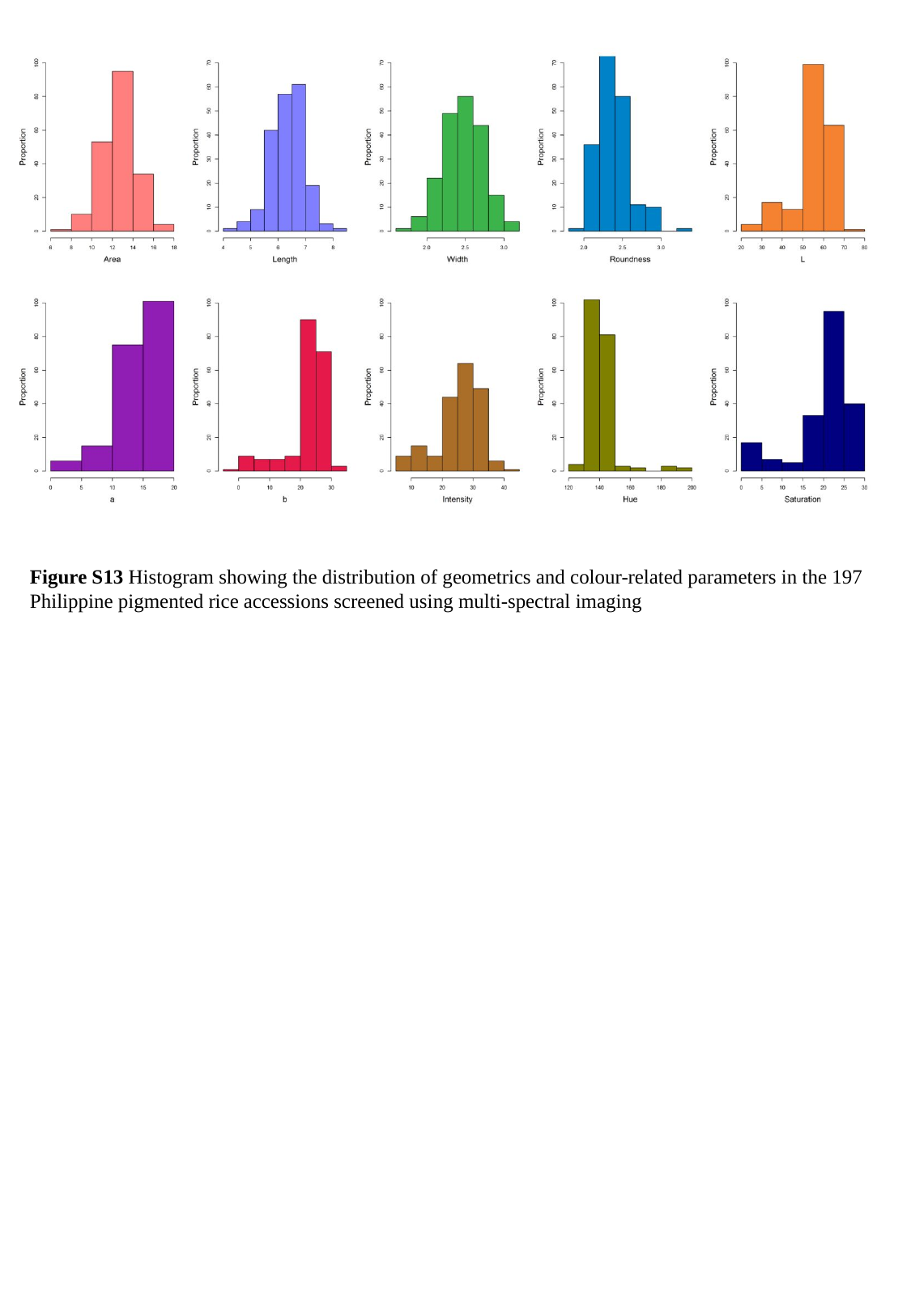

Figure S13 Histogram showing the distribution of geometrics and colour-related parameters in the 197 Philippine pigmented rice accessions screened using multi-spectral imaging

## Slide 13
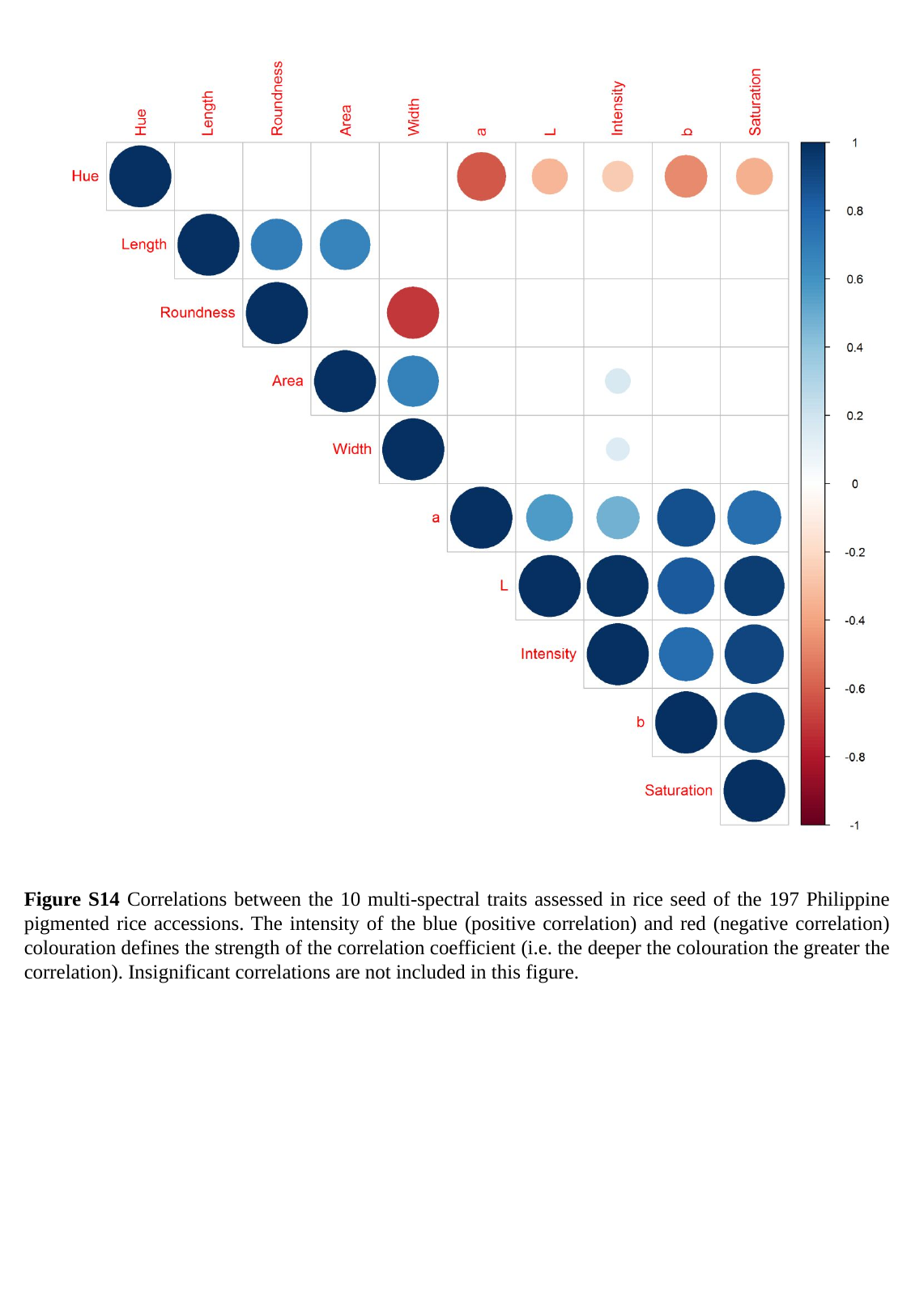

Figure S14 Correlations between the 10 multi-spectral traits assessed in rice seed of the 197 Philippine pigmented rice accessions. The intensity of the blue (positive correlation) and red (negative correlation) colouration defines the strength of the correlation coefficient (i.e. the deeper the colouration the greater the correlation). Insignificant correlations are not included in this figure.

## Slide 14
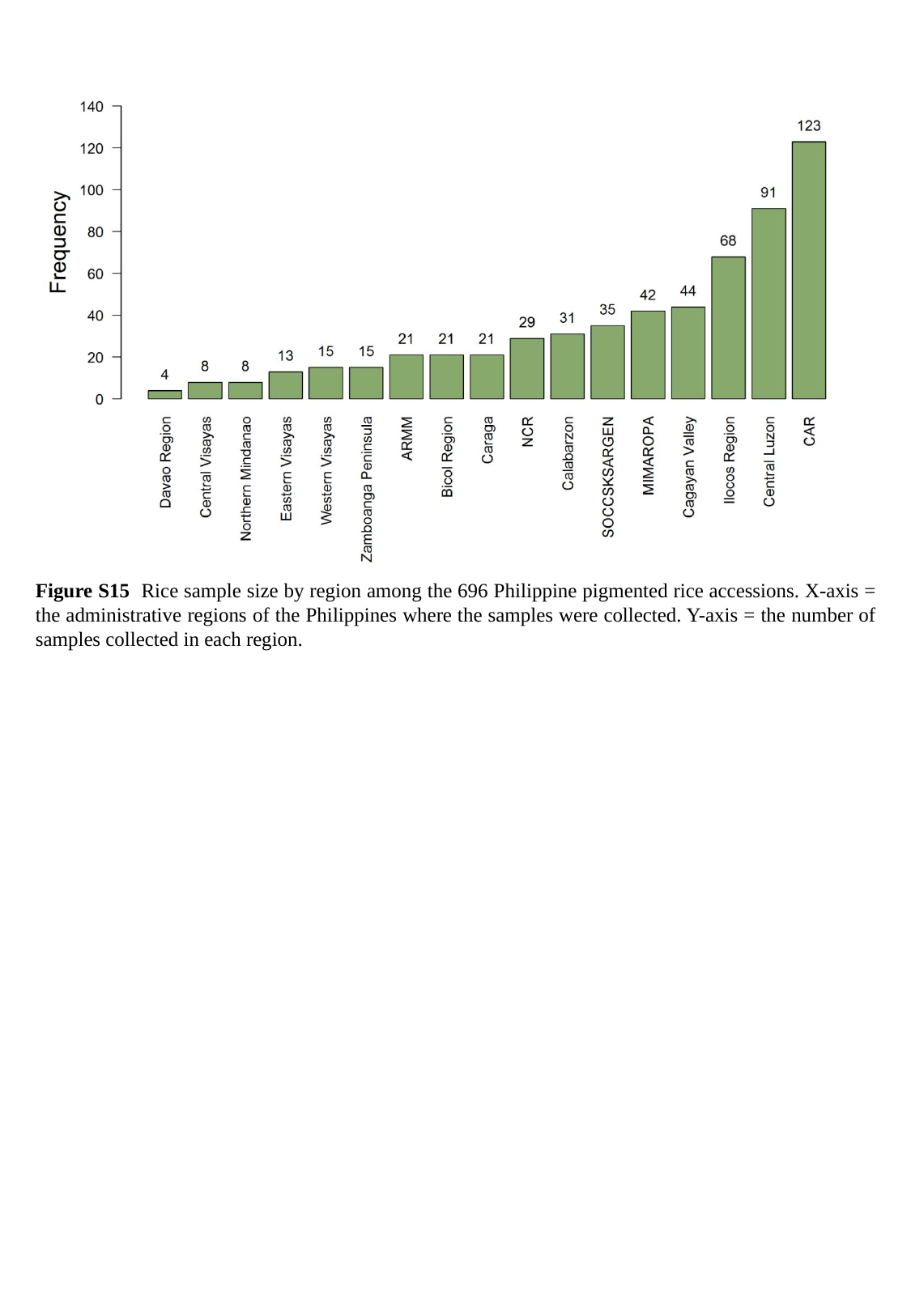

Figure S15 Rice sample size by region among the 696 Philippine pigmented rice accessions. X-axis = the administrative regions of the Philippines where the samples were collected. Y-axis = the number of samples collected in each region.
