# Supplementary material for: Exploring the genetic diversity within traditional Philippine pigmented Rice
Source: Rice (N Y). 2019 Apr 30;12:27. doi: 10.1186/s12284-019-0281-2 (PMC6491523; doi:10.1186/s12284-019-0281-2)
Supplement: Supplementary file 3 — Table S9. Philippine pigmented rice accessions allocated to redefined geographical regions. Table S10. Summary statistics of genetic diversity indicators across regions comparing the Philippine pigmented rice accessions that belong to the indica and japonica accessions. Table S11. Analysis of molecular variance of the Philippine pigmented rice accessions after removing the admix accessions. Table S12. Unique set of SNP markers that distinguishes individual accessions within the 307 core collection of Philippine pigmented rice accessions. Table S13. Summary of Philippine pigmented rice accessions carrying the 14-bp deletion within the rice Rc locus. (DOCX 43 kb) [file 12284_2019_281_MOESM3_ESM.docx]

**Table S9** Philippine pigmented rice accessions allocated to redefined geographical regions

| **Region** | **Regional designation** | **New defined Regions** | **Sample size** | ***admix*** | ***indica*** | ***japonica*** |
| --- | --- | --- | --- | --- | --- | --- |
| **NCR** | NCR | NCR | 15 | 0 | 14 | 1 |
| **CAR** | CAR | CAR | 45 | 4 | 22 | 19 |
| **Ilocos Region** | Region I | Ilocos Region | 38 | 1 | 33 | 4 |
| **Cagayan Valley** | Region II | Cagayan Valley | 11 | 1 | 2 | 8 |
| **Central Luzon** | Region III | Central Luzon | 44 | 3 | 14 | 27 |
| **Calabarzon** | Region IVA | Calabarzon | 19 | 0 | 6 | 13 |
| **MIMAROPA** | Region IVB | MIMAROPA | 32 | 0 | 5 | 27 |
| **Bicol Region** | Region V | Bicol Region | 11 | 0 | 7 | 4 |
| **Western Visayas** | Region VI | Visayas | 19 | 1 | 8 | 10 |
| **Central Visayas** | Region VII |  |  |  |  |  |
| **Eastern Visayas** | Region VIII |  |  |  |  |  |
| **Zamboanga Peninsula** | Region IX | Mindanao1 | 31 | 0 | 6 | 25 |
| **Northern Mindanao** | Region X |  |  |  |  |  |
| **ARMM** | ARM |  |  |  |  |  |
| **SOCCSKSARGEN** | Region XII | Mindanao2 | 20 | 1 | 6 | 13 |
| **Davao Region** | Region XI | Mindanao3 | 22 | 0 | 9 | 13 |
| **Caraga** | Region XIII |  |  |  |  |  |

**Table S10** Summary statistics of genetic diversity indicators across regions comparing the Philippine pigmented rice accessions that belong to the *indica* and *japonica* accessions

|  |  | **A** | **%** | **Ar**±SD | **Ho**±SD | **He**±SD | **FIS** | **FIS_Low** | **FIS_High** |
| --- | --- | --- | --- | --- | --- | --- | --- | --- | --- |
| ***indica*** | Bicol Region | 2523 | 91.37 | 1.5±0.40 | 0.07±0.11 | 0.24±0.20 | 0.72 | 0.48 | 0.90 |
|  | Calabarzon | 2443 | 88.77 | 1.45±0.40 | 0.01±0.08 | 0.23±0.20 | 0.94 | 0.89 | 0.97 |
|  | CAR | 2604 | 94.01 | 1.45±0.37 | 0.03±0.08 | 0.21±0.19 | 0.87 | 0.71 | 0.96 |
|  | Central Luzon | 2529 | 91.57 | 1.45±0.38 | 0.02±0.08 | 0.22±0.20 | 0.93 | 0.89 | 0.96 |
|  | Ilocos Region | 2565 | 92.74 | 1.44±0.37 | 0.01±0.08 | 0.22±0.19 | 0.95 | 0.94 | 0.96 |
|  | MIMAROPA | 2337 | 85.32 | 1.41±0.40 | 0.01±0.08 | 0.21±0.21 | 0.95 | 0.86 | 0.98 |
|  | Mindanao1 | 2387 | 86.95 | 1.44±0.41 | 0.01±0.07 | 0.22±0.21 | 0.96 | 0.94 | 0.97 |
|  | Mindanao2 | 2424 | 88.15 | 1.46±0.40 | 0.03±0.09 | 0.22±0.21 | 0.88 | 0.76 | 0.97 |
|  | Mindanao3 | 2462 | 89.39 | 1.46±0.40 | 0.01±0.06 | 0.23±0.21 | 0.97 | 0.96 | 0.97 |
|  | NCR | 2570 | 92.9 | 1.51±0.39 | 0.06±0.1 | 0.24±0.20 | 0.74 | 0.49 | 0.94 |
|  | Visayas | 2453 | 89.1 | 1.44±0.39 | 0.01±0.07 | 0.22±0.20 | 0.96 | 0.94 | 0.97 |
|  | **Means** | **2481.55±8.67** | **90.02±83.42** | **1.46±0.03** | **0.025±0.02** | **0.22±0.01** | **0.9±0.09** | **0.81±0.18** | **0.96±0.02** |
|  |  |  |  |  |  |  |  |  |  |
| ***japonica*** | Bicol Region | 2115 | 86.46 | 1.29±0.38 | 0.01±0.08 | 0.16±0.21 | 0.93 | 0.85 | 1.0 |
|  | Cagayan Valley | 2274 | 91.63 | 1.31±0.35 | 0.01±0.08 | 0.17±0.20 | 0.93 | 0.90 | 0.94 |
|  | Calabarzon | 2351 | 94.14 | 1.35±0.36 | 0.01±0.08 | 0.19±0.20 | 0.94 | 0.93 | 0.95 |
|  | CAR | 2396 | 95.61 | 1.33±0.34 | 0.01±0.07 | 0.18±0.19 | 0.96 | 0.95 | 0.96 |
|  | Central Luzon | 2412 | 96.13 | 1.36±0.36 | 0.01±0.07 | 0.2±0.20 | 0.95 | 0.94 | 0.96 |
|  | Ilocos Region | 2150 | 87.6 | 1.29±0.37 | 0.01±0.07 | 0.16±0.21 | 0.95 | 0.92 | 0.99 |
|  | MIMAROPA | 2429 | 96.68 | 1.36±0.36 | 0.01±0.07 | 0.2±0.20 | 0.95 | 0.92 | 0.96 |
|  | Mindanao1 | 2424 | 96.52 | 1.37±0.36 | 0.02±0.09 | 0.2±0.20 | 0.92 | 0.88 | 0.94 |
|  | Mindanao2 | 2341 | 93.82 | 1.35±0.37 | 0.02±0.09 | 0.18±0.20 | 0.89 | 0.78 | 0.95 |
|  | Mindanao3 | 2340 | 93.78 | 1.34±0.36 | 0.01±0.07 | 0.19±0.20 | 0.95 | 0.92 | 0.96 |
|  | Visayas | 2220 | 89.88 | 1.28±0.34 | 0.01±0.08 | 0.15±0.19 | 0.93 | 0.89 | 0.95 |
|  | **Means** | **2313.82±110.16** | **92.93±3.57** | **1.33±0.03** | **0.01±0.004** | **0.18±0.02** | **0.94±0.02** | **0.90±0.05** | **0.96±0.02** |

Total number of alleles observed across SNP marker loci (A). Total observed alleles per locus as a percentage of population sample (%). Mean allele richness (Ar). Observed heterozygosity across loci (Ho). Expected heterozygosity across loci (He). Inbreeding coefficient (FIS). Standard Deviations (SD).

**Table S11** Analysis of molecular variance of the Philippine pigmented rice accessions after removing the admix accessions

|  |  | **Degrees of freedom** | **Sum of**  **Squares** | **Mean sum of squares** | **Estimated variance** | **% of variation** |
| --- | --- | --- | --- | --- | --- | --- |
| **Subspecies**  **(no admix)** | Between rice varietal types | 1 | 52621.97 | 52621.97 | 178.71 | 50.14 |
|  | Between accessions within varietal types | 294 | 100783.15 | 342.80 | 165.06 | 46.31 |
|  | Within rice accessions | 296 | 3755.64 | 12.69 | 12.69 | 3.56 |
|  | **Total variation** | **591** | **157160.76** | **265.92** | **356.45** | **100** |
|  |  |  |  |  |  |  |
| ***Indica*** | Between rice varietal types | 10 | 6946.71 | 694.67 | 14.90 | 7.31 |
|  | Between accessions within varietal types | 119 | 42845.28 | 360.04 | 171.22 | 84.05 |
|  | Within rice accessions | 130 | 2287.5 | 17.60 | 17.60 | 8.63 |
|  | **Total variation** | **259** | **52079.49** | **201.08** | **203.71** | **100** |
|  |  |  |  |  |  |  |
| ***Japonica*** | Between rice varietal types | 10 | 6634.35 | 663.43 | 12.58 | 7.49 |
|  | Between accessions within varietal types | 152 | 45887.01 | 301.89 | 146.47 | 87.18 |
|  | Within rice accessions | 163 | 1459 | 8.95 | 8.95 | 5.33 |
|  | **Total variation** | **325** | **53980.36** | **166.09** | **168.00** | **100** |

**Table S12** Unique set of SNP markers that distinguishes individual accessions within the 307 core collection of Philippine pigmented rice accessions

| **Name** | **Chromosome** | **Position** | **Alleles** |
| --- | --- | --- | --- |
| 1511115 | 2 | 5624639 | T/C |
| 2560888 | 3 | 3569876 | T/C |
| id3005216 | 3 | 10093744 | A/G |
| id3010971 | 3 | 25144373 | A/G |
| 4566321 | 4 | 26420848 | G/A |
| 4602299 | 4 | 27851291 | T/G |
| id5006793 | 5 | 17047998 | G/T |
| 5754154 | 5 | 27842681 | C/T |
| 6986149 | 7 | 1060217 | C/T |
| 7370424 | 7 | 11757207 | G/A |
| id7002749 | 7 | 17422851 | A/G |
| c7p27670416 | 7 | 27670417 | A/G |
| 8608433 | 8 | 15666434 | A/G |
| 9422380 | 9 | 8851945 | C/T |
| 10705056 | 10 | 18877199 | G/A |
| 11982569 | 11 | 28437127 | A/G |
| 12010072 | 12 | 246313 | A/G |
| id12008328 | 12 | 23828922 | C/T |
| 12973423 | 12 | 23841123 | C/A |
| 13022390 | 12 | 25491650 | C/A |

**Table S13 Summary of Philippine pigmented rice accessions carrying the 14-bp deletion within the rice *Rc* locus**

|  |  | **Red** | **Purple** | **Variable purple** | **Mix (red and white)** | **Mix ( red and variable purple)** | **white** | **Total** |
| --- | --- | --- | --- | --- | --- | --- | --- | --- |
| **admix** | Ambiguous | 3 | - |  | - | - | - | 3 |
|  | Deletion | - | - | 1 | - | - | - | 1 |
|  | No deletion | 4 | - | 1 | - | - | - | 5 |
|  | **Total** | **7** | **-** | **2** | **-** | **-** | **-** | **9** |
|  |  |  |  |  |  |  |  |  |
| ***indica*** | Ambiguous | 3 | - |  | - | - | - | 3 |
|  | Deletion | 12 | 4 | 1 | 1 | - | 2 | 20 |
|  | No deletion | 91 | 1 | 5 | 1 | - | - | 98 |
|  | **Total** | **106** | **5** | **6** | **2** | - | 2 | **121** |
|  |  |  |  |  |  |  |  |  |
| ***japonica*** | Ambiguous | 1 | - | - | - | - | - | 1 |
|  | Deletion | 3 | 4 | 2 | 1 | - | 1 | 8 |
|  | No deletion | 75 | 7 | 13 | 1 | 1 | 2 | 99 |
|  | **Total** | **79** | **11** | **15** | **2** | **1** | **3** | **111** |
